# Supplementary material for: FtsN maintains active septal cell wall synthesis by forming a processive complex with the septum-specific peptidoglycan synthases in E. coli
Source: Nat Commun. 2022 Sep 30;13:5751. doi: 10.1038/s41467-022-33404-8 (PMC9525312; doi:10.1038/s41467-022-33404-8)
Supplement: Supplementary file 1 — Supplementary Information [file 41467_2022_33404_MOESM1_ESM.pdf]

Supplementary Information

FtsN maintains active septal cell wall synthesis by forming a processive complex with the septum-specific peptidoglycan synthases in *E. coli*

Zhixin Lyu[1], Atsushi Yahashiri[2], Xinxing Yang[1,3], Joshua W. McCausland[1], Gabriela M. Kaus[2], Ryan McQuillen[1], David S. Weiss[2*], Jie Xiao[1*]

[1]Department of Biophysics and Biophysical Chemistry, Johns Hopkins School of Medicine, Baltimore, MD 21205, USA.

[2]Department of Microbiology and Immunology, University of Iowa Carver College of Medicine, Iowa City, IA 52242, USA.

[3]Current address: The Chinese Academy of Sciences Key Laboratory of Innate Immunity and Chronic Disease, School of Basic Medical Sciences, Division of Life Sciences and Medicine, University of Science and Technology of China, Hefei, China.

*Corresponding authors: david-weiss@uiowa.edu, xiao@jhmi.edu

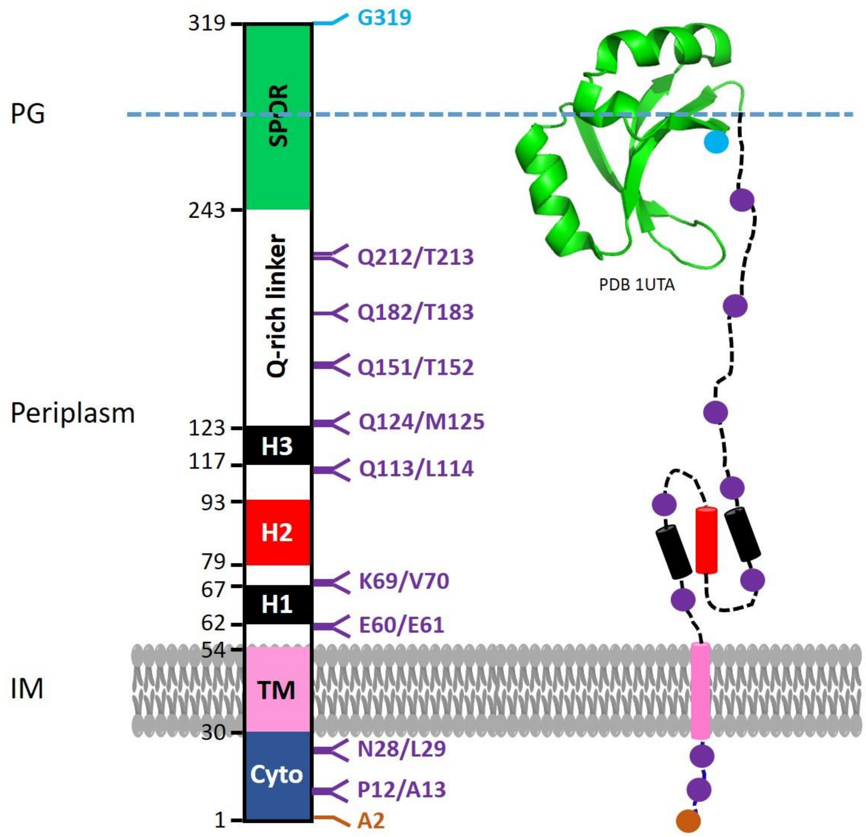

Supplementary Fig. 1. Sites used for mNG fusions to FtsN.

mNG was fused to the N-terminus (orange), C-terminus (cyan) or inserted at internal positions (purple) of FtsN as shown by the amino acid numbers (left) and corresponding dots (right). The domain structure of FtsN is illustrated with different colors, which are the N-terminal cytoplasmic domain ($\text{FtsN}^{\text{Cyto}}$, blue), the transmembrane domain (FtsN^{TM}, pink), the periplasmic essential domain (FtsN^{E}) containing helices H1 (black), H2 (red), and H3 (black) with H2 being essential for FtsN function in cell division, and the C-terminal SPOR domain (PDB 1UTA[1], $\text{FtsN}^{\text{SPOR}}$, green). Numbers by the left side of the domain regions refer to the amino acid range of different domains.

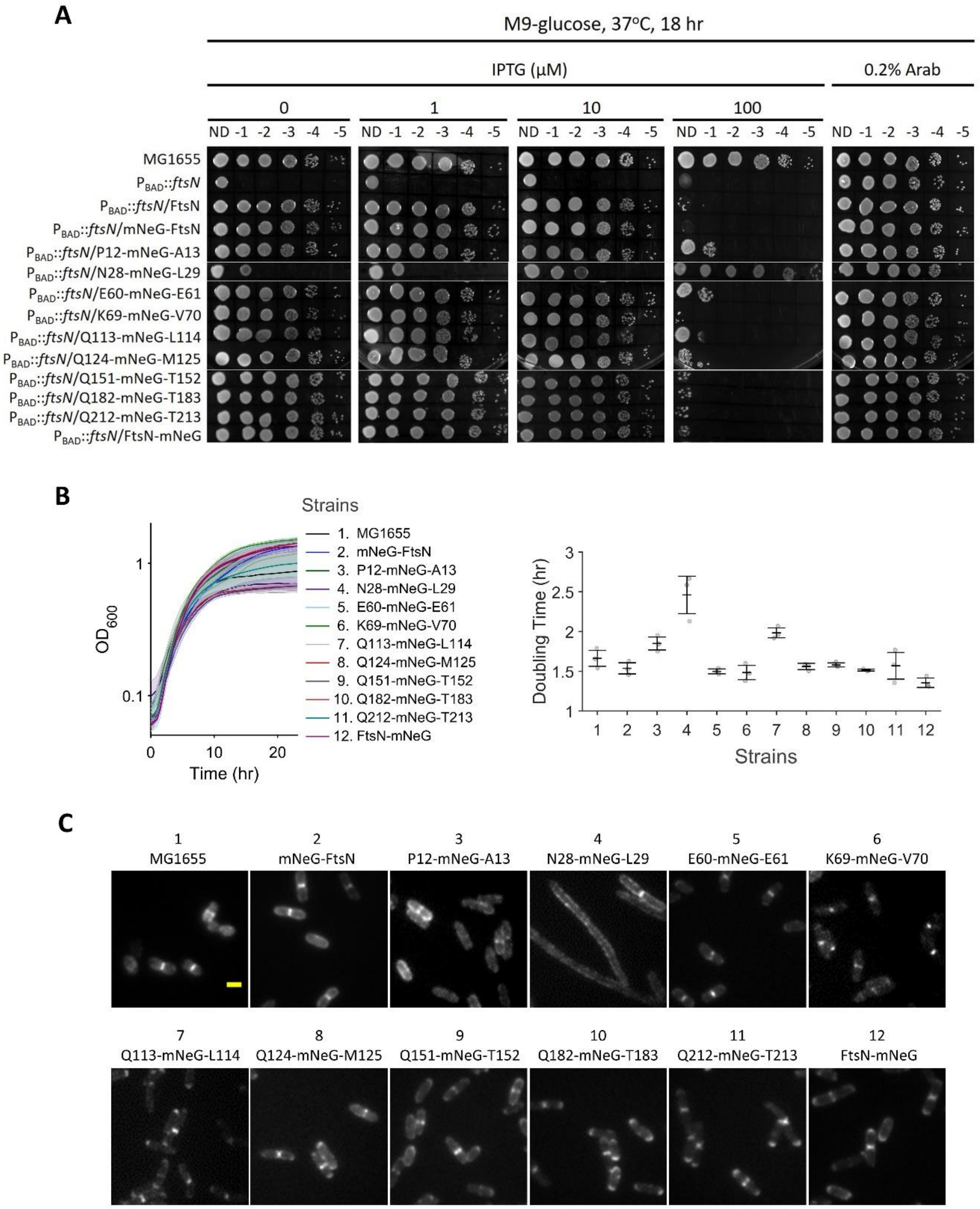

Supplementary Fig. 2. Characterization of mNG-fused FtsN constructs.

(A) To test for complementation on plates, cultures were serially diluted 10-fold, spotted onto M9-glucose plates containing increasing IPTG concentrations, and incubated at 37°C for 18 hrs. The protein expressed under IPTG control is indicated for each strain. M9-glucose plates containing 0.2% Arab to express chromosomal wildtype (WT) FtsN in each strain background serve as the positive control. Data were combined from two

experiments. ND: no dilution. (B) Growth curves of MG1655 and FtsN-depletion strains expressing various fusions of mNG to FtsN in M9-glucose minimal media with no IPTG (i.e., leaky expression) at 30°C (mean ± *s.e.m.*, n = 3 biological replicates). The doubling time was calculated from the growth curves (mean ± standard deviation, n = 3 biological replicates). The numbers on the x-axis are the strain numbers, which correspond to the strains listed left sequentially. (C) Septal localization of various mNG fusions to FtsN. MG1655 cells (no FtsN fusion) were imaged by immunofluorescence staining. Cells from other strains were grown in M9-glucose minimal media without induction. Experiment was repeated three times with similar results. Scale bar, 1 µm. Source data are provided as a Source Data file.

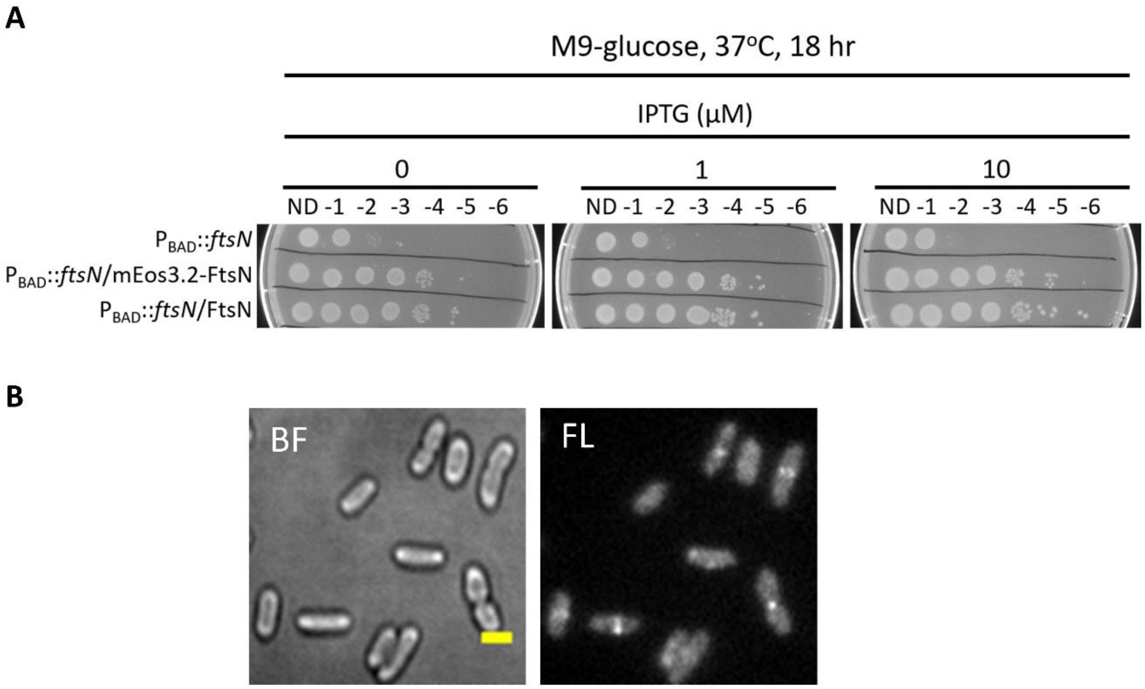

Supplementary Fig. 3. Characterization of the mEos3.2-FtsN fusion strain. (A) To test for complementation on plates, cultures were serially diluted 10-fold, spotted onto M9-glucose plates containing increasing IPTG concentrations, and incubated at 37°C for 18 hrs. The protein expressed under IPTG control is indicated for the strain. ND: no dilution. (B) Integrated green fluorescence images of mEos3.2-FtsN (Strain EC4443 in Supplementary Table 1) were acquired by excitation at 488-nm without UV activation. Please note that mEos3.2 is not as bright as GFP or mNG when serving as a green fluorescent protein. Cells were grown in M9-glucose minimal media without induction. Experiment was repeated three times with similar results. Scale bar, 1 μm.

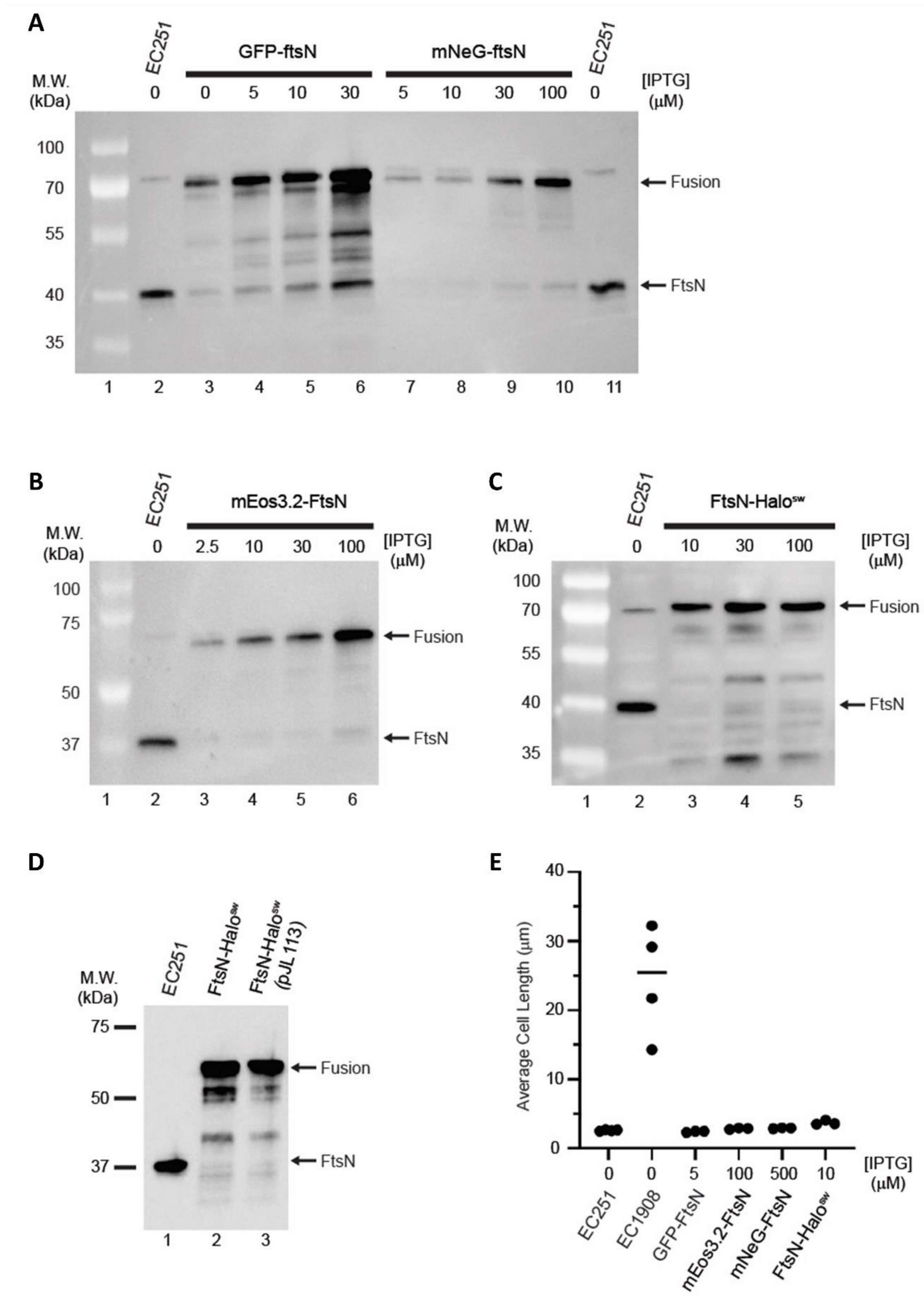

A
EC251
GFP-ftsN
mNeG-ftsN
EC251
M.W. (kDa)
[IPTG] (μM)
0 0 5 10 30 5 10 30 100 0
100
70
55
40
35
Fusion
FtsN
1 2 3 4 5 6 7 8 9 10 11
B
EC251
mEos3.2-FtsN
M.W. (kDa)
0 2.5 10 30 100
[IPTG] (μM)
100
75
50
37
Fusion
FtsN
1 2 3 4 5 6
C
EC251
FtsN-Halo^sw
M.W. (kDa)
0 10 30 100
[IPTG] (μM)
100
70
55
40
35
Fusion
FtsN
1 2 3 4 5
D
M.W. (kDa)
EC251
FtsN-Halo^sw
FtsN-Halo^sw (pJL113)
75
50
37
Fusion
FtsN
1 2 3
E
Average Cell Length (μm)
40
30
20
10
0
0 0 5 100 500 10
[IPTG] (μM)
EC251
EC1908
GFP-FtsN
mEos3.2-FtsN
mNeG-FtsN
FtsN-Halo^sw

Supplementary Fig. 4. Validation of FtsN fusions with mEos3.2, GFP, mNG and Halo integrated into a chromosomal phage attachment site in an FtsN-depletion strain background (EC1908).

(A-D) Western blots with anti-$FtsN^{peri}$ sera showing the expression levels and stability of fusion proteins at different induction conditions. For all imaging experiments, the IPTG induction level was as shown in (E). Size markers are indicated to the left of each blot. Blots are representative of at least two trials. (E) Average cell length from 4 trials with ≥ 200 cells measured per trial. Cells were grown at room temperature in M9-glucose with IPTG as indicated to OD_{600} ~0.35 before sampling for Western blotting or fixing for microscopy. The strains shown in (A-C) and (E) are EC251 (WT FtsN), EC1908 (P_{BAD}::*ftsN* for FtsN depletion), EC4240 (GFP-FtsN), EC4443 (mEos3.2-FtsN), EC4564 (mNG-FtsN), and EC5234 (FtsN-$Halo^{SW}$, insertion at E60-E61). Strains shown in (D) are EC251, EC5234, and EC5606. See more strain details in Supplementary Table 1. Source data are provided as a Source Data file.

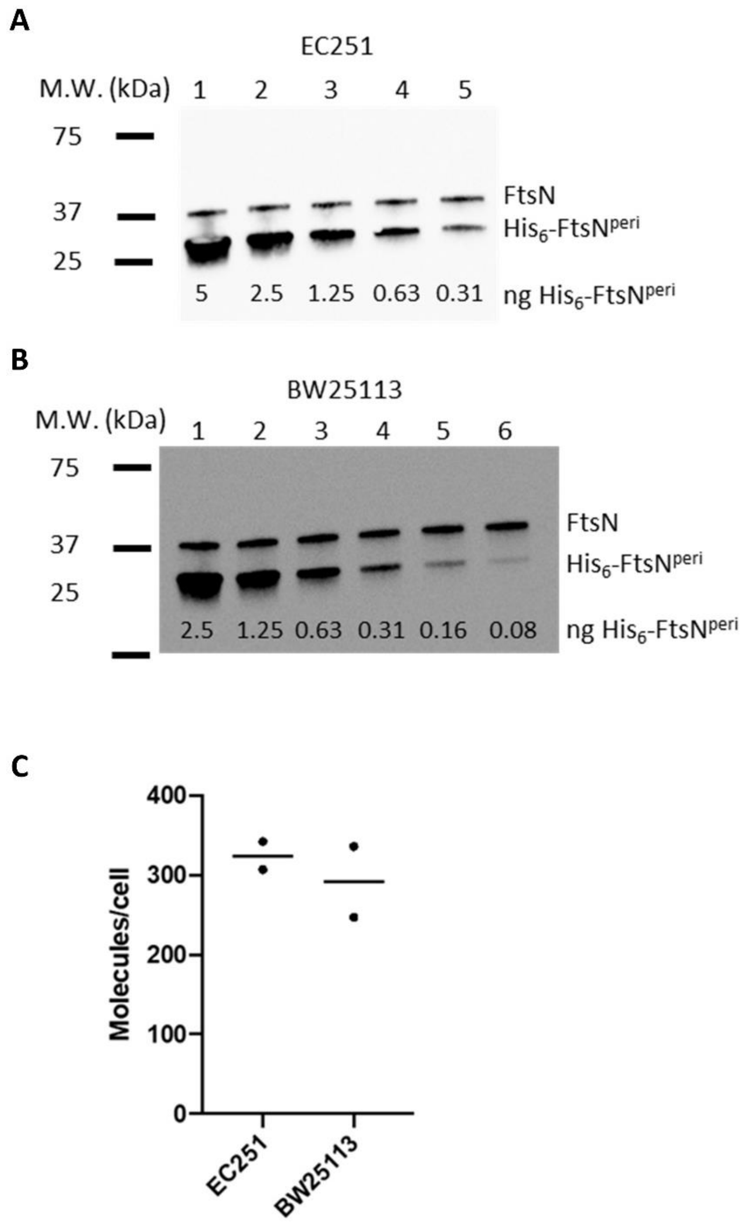

Supplementary Fig. 5. Quantitation of FtsN copy number in MG1655 and BW25113 strains.

(A and B) Representative Western blots using anti-FtsN^{peri} sera. The amount of FtsN in 1.9 x 10^7 cells of EC251 (A) and 1.7 x 10^7 cells of BW25113 (B) was compared to a standard curve generated by diluting purified His_6-FtsN periplasmic domain into the cell extracts. In the blots shown, the signal intensity of native FtsN in EC251 and BW25113 corresponded to 0.35 and 0.38 ng of His_6-FtsN^{peri}, respectively. (C) Average number of FtsN molecules per cell (bar) in two experiments (dots) for each strain. This calculation takes into account differences in molecular mass (FtsN = 35.793 kDa, His_6-FtsN^{peri} = 31.856 kDa) and the number of cells loaded in each lane. Source data are provided as a Source Data file.

A

$$\rho(r) = A_1\left(\frac{1}{\sqrt{\pi}\sigma_{res}}\right)e^{\frac{-r^2}{4\sigma_{res}^2}} + A_2\left(\frac{1}{\sqrt{2\pi\omega^2}}\right)e^{\frac{-(r-d_c)^2}{2\omega^2}}$$

B

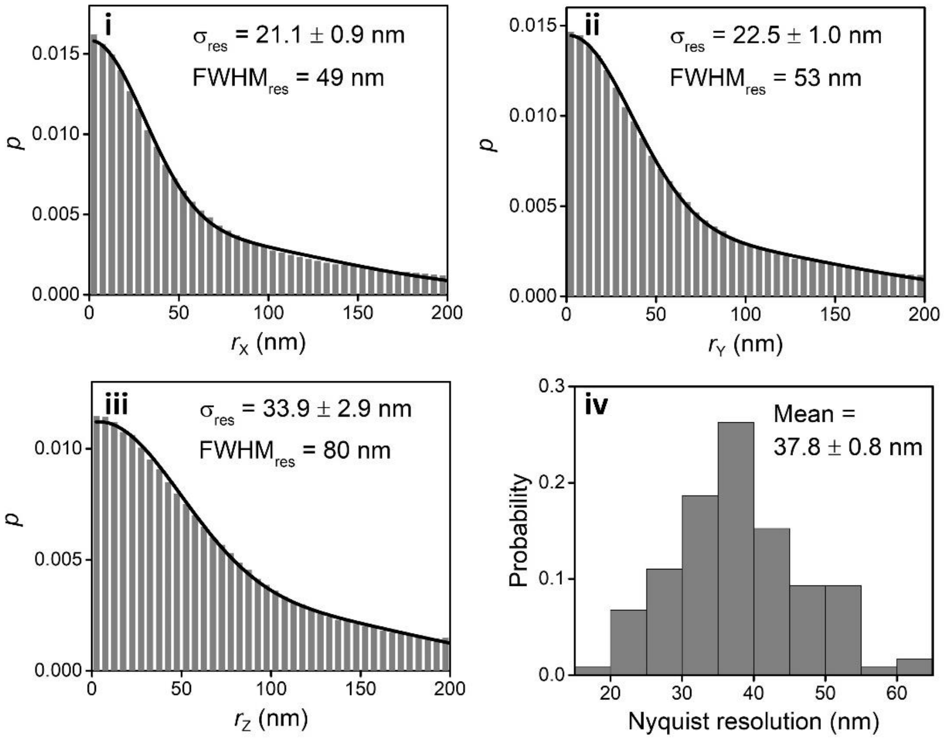

C

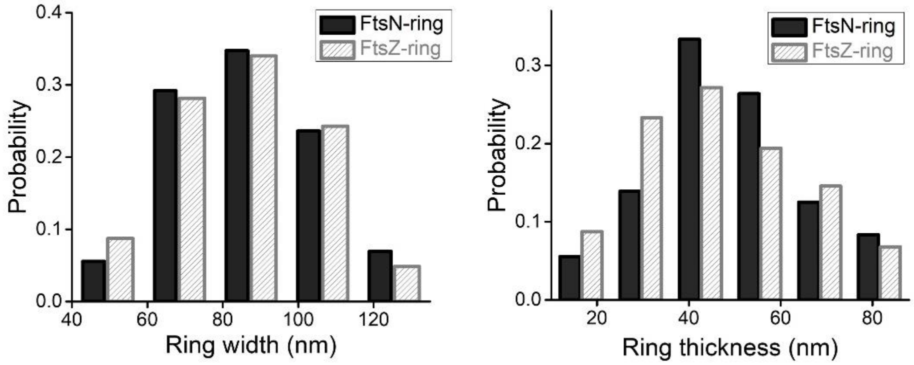

Supplementary Fig. 6. Spatial resolution of 3D SMLM imaging and dimensions of FtsN- and FtsZ-rings.

(A) Equation describing the distribution (*p*) of pair-wise distances (*r*) between nearest neighbors in adjacent frames of live-cell SMLM data[2]. The first term represents the distribution expected for repeat observations of the same molecule with localization precision σ_{res}. The second term with Gaussian parameters ω and d_c accounts for the possibility that nearest neighbors in adjacent frames may not arise from the same

molecule. (B) i-iii, Distributions of pair-wise distances between nearest neighbors in adjacent frames (gray bars) from SMLM imaging data along the x- (i), y- (ii), and z-axes (iii). Each histogram was fit using the equation in (A) to generate the black fitted curves. The achieved localization precision (σ_{res}) and spatial resolution (expressed as $FWHM_{res}$) determined from these fitted curves are displayed as insets. iv: Distribution of Nyquist resolution which was calculated as previously described[3]. (C) Distributions of resolution-deconvolved width (left) and thickness (right) of FtsN-rings (black, n = 72 cells) and FtsZ-rings (gray, n = 103 cells, data from a previous work[4]). There are no significant differences in the dimensions between the FtsN- and FtsZ-rings. Source data are provided as a Source Data file.

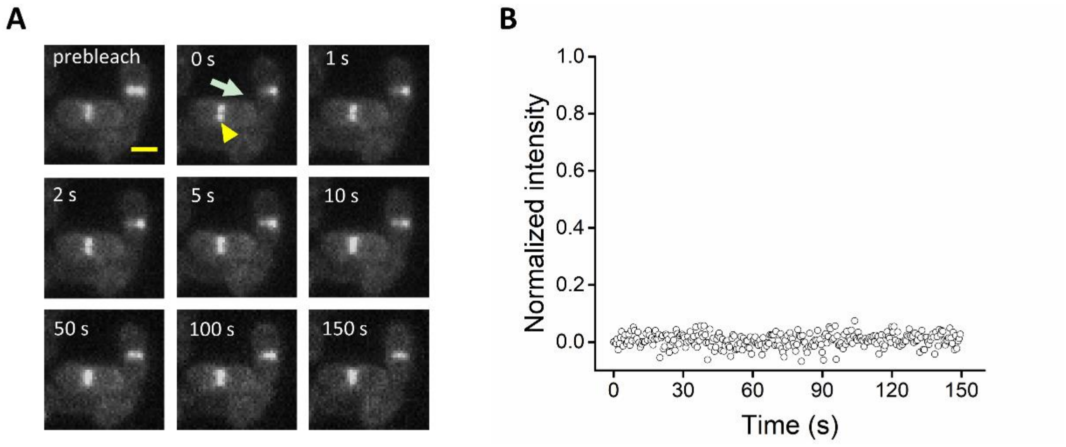

Supplementary Fig. 7. FRAP analysis of FtsN.
(A) A representative FRAP imaging sequence showing the recovery of fluorescence after the photobleaching of half of the FtsN-ring (cyan arrow, Strain EC4240 in Supplementary Table 1). An adjacent cell without photobleaching serves as the control (yellow arrowhead). Scale bar, 1 µm. (B) Mean FRAP recovery curve of FtsN from adjacent control cells (gray, n = 6 cells) showed no fluorescent intensity changed on the time scale of experiment. The global photobleaching was corrected by using the fluorescent intensity outside the septum. The FRAP curve was close to 0 after subtracting the first acquisition. See more details in the Methods section. Source data are provided as a Source Data file.

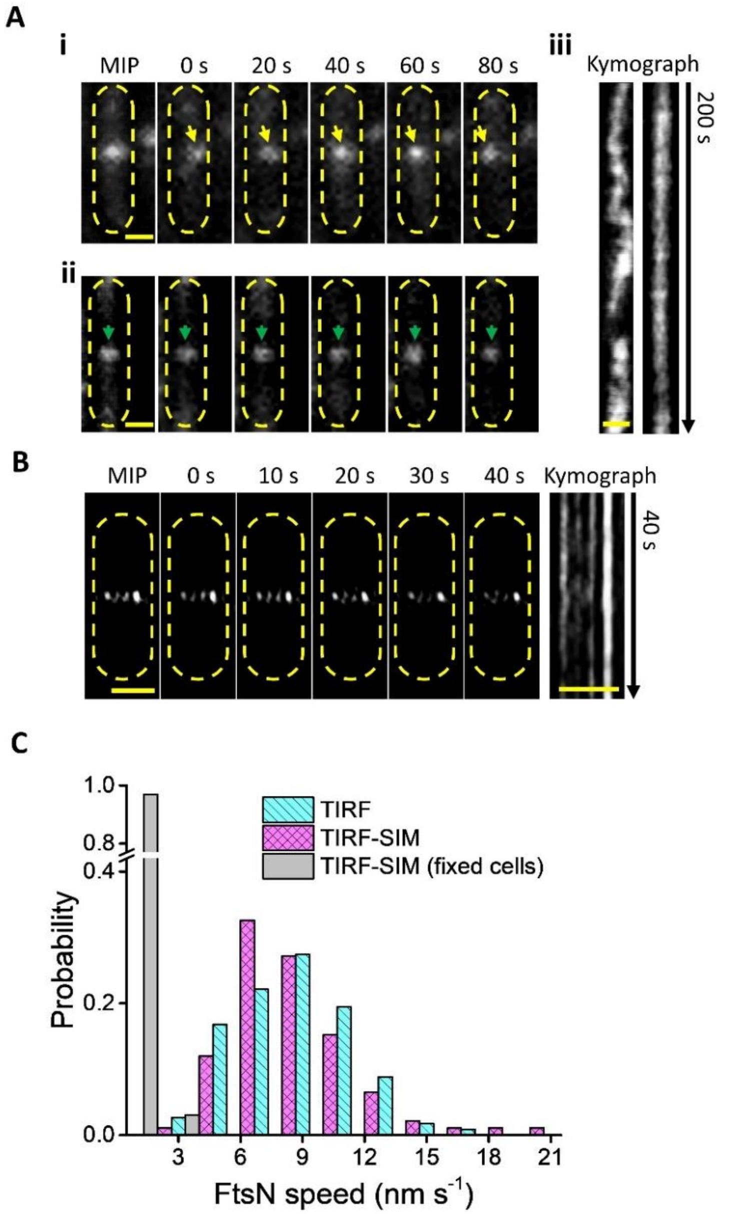

Supplementary Fig. 8. FtsN clusters exhibit slow, directional motions.
(A) i-ii, Maximum intensity projection (MIP) and montages from TIRF time-lapse imaging of two cells in which a cluster is moving (i, yellow arrow) or immobile (ii, green arrow). Scale bars, 500 nm. iii, Kymographs of the cells in (i and ii) computed from the intensity along a line across the midcell are shown. Scale bars, 250 nm. (B) Maximum intensity projection (MIP) and montages from TIRF-SIM time-lapse imaging of a fixed cell in which the clusters are immobile (left). Kymograph computed from the intensity along a line across the midcell (right). Scale bars, 500 nm. (C) Distributions of FtsN clusters' moving speeds as measured from the kymographs (TIRF, cyan, v = 8.6 ± 0.3 nm s^{-1}, μ ± *s.e.m.*, n = 113 clusters; TIRF-SIM, magenta, v = 8.8 ± 0.3 nm s^{-1}, μ ± *s.e.m.*, n = 92 clusters; TIRF-SIM (fixed cells), gray, v = 0.57 ± 0.07 nm s^{-1}, μ ± *s.e.m.*, n = 65 clusters). Source data are provided as a Source Data file.

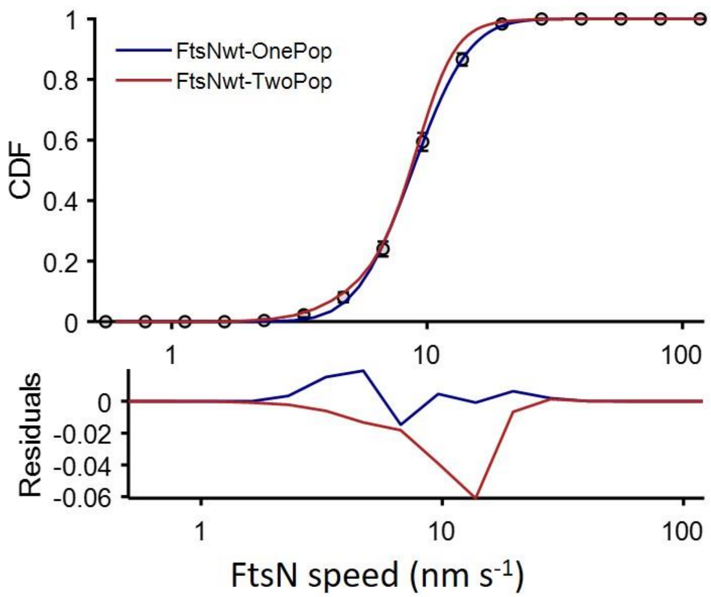

Supplementary Fig. 9. Single- or double-population fitting of the cumulative probability density (CDF) of FtsN's directional moving speed distribution. Cumulative probability density (CDF) curve of the directional moving speed of FtsN molecules in WT MG1655 cells (black dots) was best fit by a single- (blue curve) instead of a double- (red curve) population (empirically using log-normal distribution to describe the long tail), as indicated by the residuals below. Error bars indicate *s.e.m.* from bootstrapping the CDF fitting 200 times.

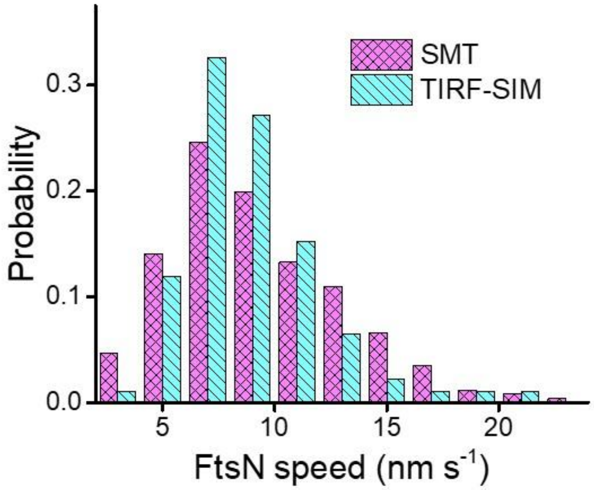

Supplementary Fig. 10. Comparison of the speed distributions from SMT and TIRF-SIM.

Distributions of single FtsN molecules' moving speeds from SMT (magenta, n = 256) and single FtsN clusters' moving speeds from TIRF-SIM (cyan, n = 92). The p-value of the two-sample Kolmogorov-Smirnov (K-S) test for the two distributions is 0.15 (>0.05), indicating they are statistically identical. Source data are provided as a Source Data file.

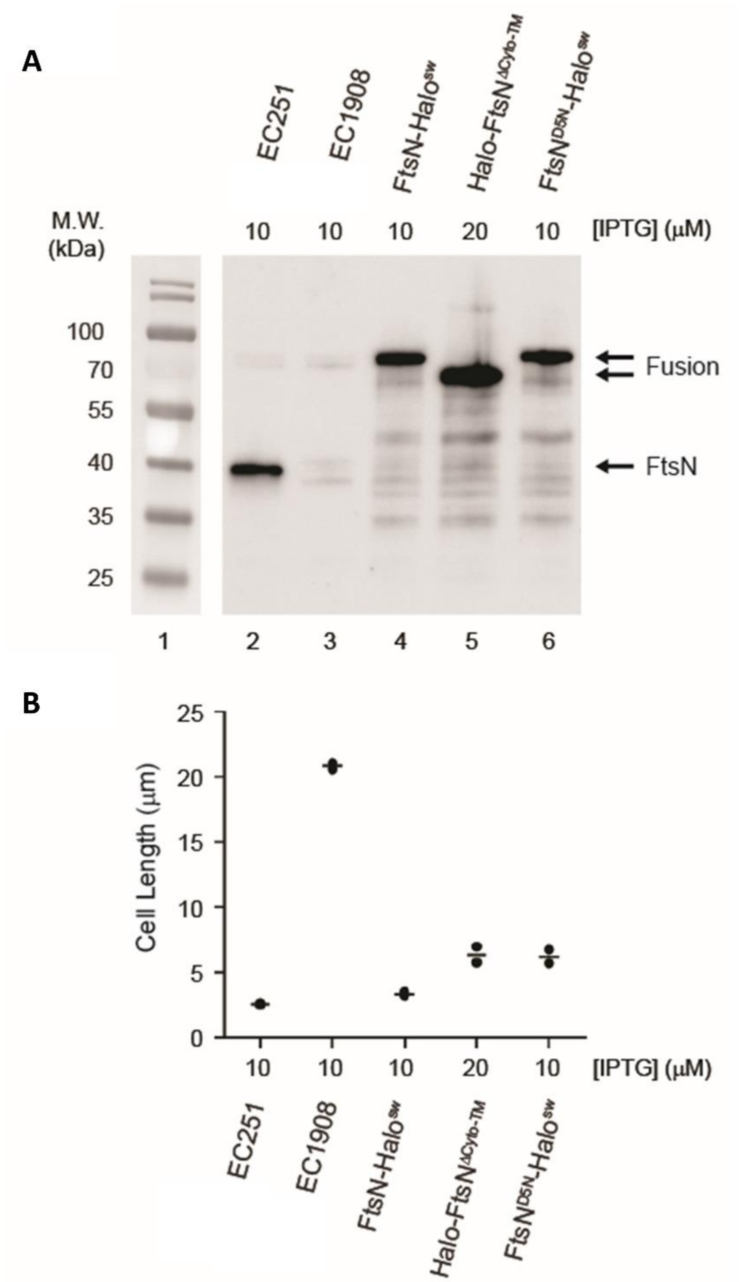

Supplementary Fig. 11. Validation of Halo sandwich fusions to mutant FtsN proteins defective in interaction with FtsA.

(A) Western blot with anti-FtsN^{peri} sera documenting fusion protein expression and effective depletion of native FtsN. Blot was deliberately overexposed to highlight the lack of residual FtsN in these strains. Blot is representative of two experiments. (B) Average cell length from two experiments with ≥ 200 cells measured per experiment. Cells were grown in M9-glucose plus IPTG as indicated. Samples were taken for Western blotting and microscopy at OD_{600} ~0.35. Strains shown are EC251 (WT), EC1908 (P_{BAD}::*ftsN*), and EC1908 derivatives that express the indicated *ftsN* fusion under control of a modified *Trc* promoter (EC5234, EC5263, and EC5271). See more strain details in Supplementary Table 1. Source data are provided as a Source Data file.

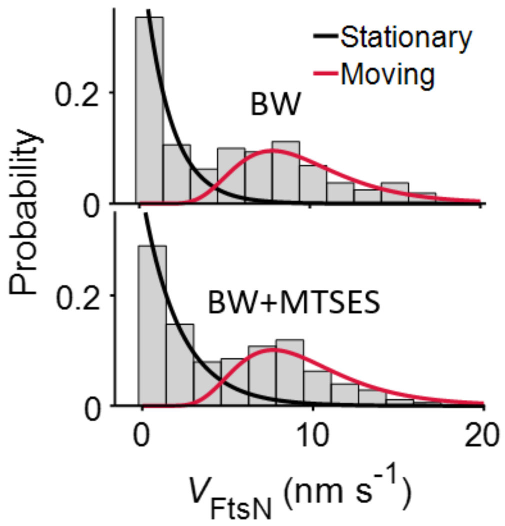

Supplementary Fig. 12. MTSES did not alter FtsN-Halo^{SW} dynamics in the BW25113 WT strain.

Speed distributions and the corresponding fit curves of the stationary (black) and moving (red) populations of single FtsN-Halo^{SW} molecules in the BW25113 WT strain in the absence (top, n = 161) or presence (bottom, n = 176) of MTSES. The identical fitted parameters shown in Supplementary Table 10 indicate that MTSES did not alter FtsN-Halo^{SW} dynamics in the BW25113 WT strain. Source data are provided as a Source Data file.

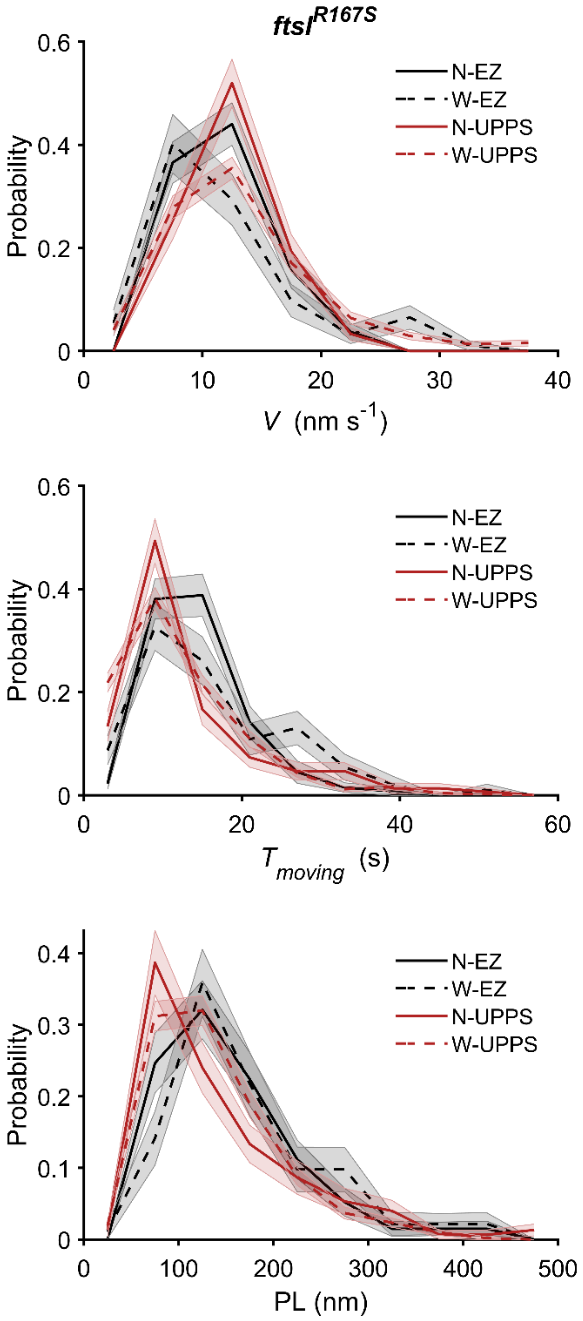

Supplementary Fig. 13. Comparison of the distributions of the speed (*V*), moving dwell time (*T*_moving), and processive running length (*PL*) of FtsN and FtsW in *ftsI*^{R167S} strain grown under the rich EZRDM growth condition and UppS overexpression condition.
The difference between the distributions of FtsN and FtsW (data from a previous work[5]) under the same condition was determined to be insignificant by the two-sample Kolmogorov-Smirnov (K-S) test. The calculated *p*-values were shown in Supplementary Table 11. The sample size of FtsN is listed in Supplementary Table 10. Both FtsN's and FtsW's histograms were bootstrapped 100 times to provide the shaded standard error bars (mean ± *s.e.m.*). Source data are provided as a Source Data file.

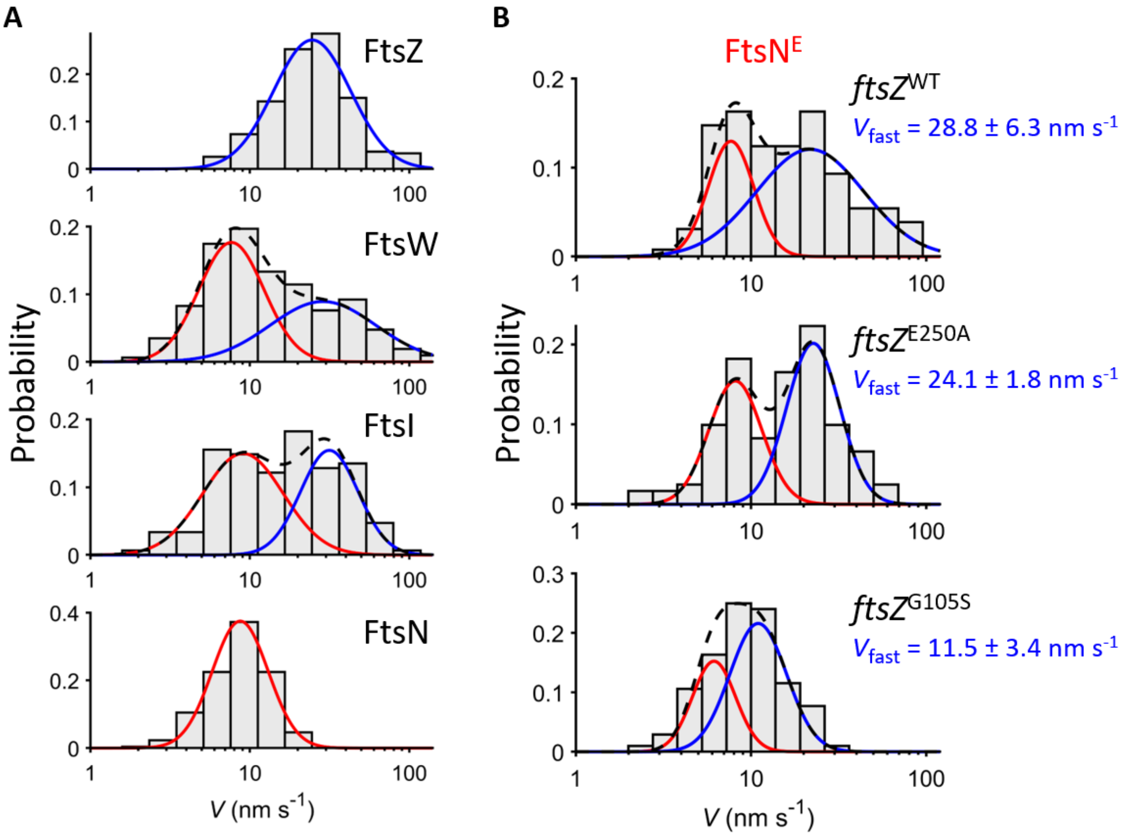

Supplementary Fig. 14. Comparison of the speed distributions of FtsZ, FtsW, FtsI, FtsN, and FtsN^{E} in different FtsZ GTPase mutant backgrounds under the same growth condition.

(A) Histograms of the speeds of FtsZ treadmilling[6], FtsW[5], FtsI[14], and FtsN in the log-normal scale were overlaid with one- or two-population fitting curves (slow-moving population in red, fast-moving population in blue and overall fit curve in black dashed lines). (B) Histograms of the speeds of FtsN^{E} in different FtsZ GTPase mutant backgrounds in the log-normal scale were overlaid with two-population fitting curves (slow-moving population in red, fast-moving population in blue and overall fit curve in black dashed lines). Strains used are JL339 (*ftsZ*^{WT}), JL421 (*ftsZ*^{E250A}) and JL422 (*ftsZ*^{G105S}), which contain *ftsB*^{E56A} and *ΔftsN::kan* in addition to the indicated *ftsZ* allele. All the speed histograms, contain only the moving population but not the stationary population, were plotted in the log-normal scale to better compare with each other. The fitted fast speed was labelled as mean ± *s.e.m.,* where errors are from 200 bootstrap samples pooled from three independent experiments. Source data are provided as a Source Data file.

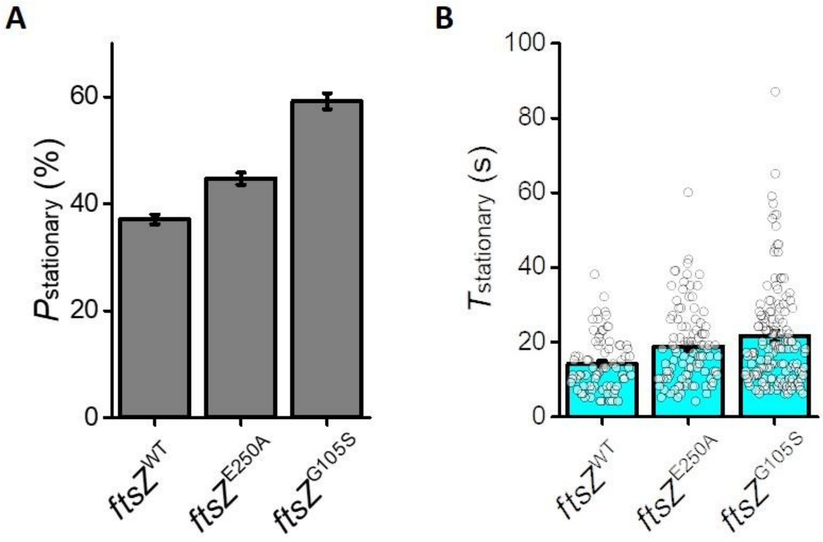

Supplementary Fig. 15. Percentage and mean dwell time of stationary FtsN^{E} molecules increased with reduced FtsZ GTPase activity.

(A) Percentage ($P_{stationary}$) of stationary FtsN^{E} molecules increased with reduced FtsZ GTPase activity. Data are presented as mean ± error, where the error is the standard deviation from 200 bootstrap samples pooled from three independent experiments. (B) Average dwell time ($T_{stationary}$) of stationary FtsN^{E} molecules increased with reduced FtsZ GTPase activity. Data are presented as mean ± *s.e.m.* overlayed with corresponding data points (shown as open circles. *ftsZ*^{WT}, n = 75; *ftsZ*^{E250A}, n = 97; *ftsZ*^{G105S}, n = 151). Source data are provided as a Source Data file.

Supplementary Table 1. Strains used in this study.

Strains	Relevant genetic markers or features	Source, reference or construction
Strains without *ftsN* fusions		
Stellar	*mcrA* Δ(*mrr-hsdRMS-mcrBC*) φ80(*lacZ*)ΔM15 Δ(*lacZYA-argF*)*U169 endA1 recA1 supE44 thi-1 gyrA96 relA1*	Takara, cloning host
MG1655	*ilvG rfb*50 *rph1*	[7]
BW25113	*rrnB3* Δ*lacZ4787 hsdR514* Δ(*araBAD)*567 Δ(*rhaBAD)568 rph-1*	[8]
OmniMAX-2 T1R	F' [*proAB*$^{+}$ *lacI*^{q} *lacZ*ΔM15 Tn*10*(Tet^{r}) Δ(*ccdAB*)] *mcrA* Δ(*mrr-hsdRMS-mcrBC*) φ80(*lacZ*)ΔM15 Δ(*lacZYA-argF*)*U169 endA1 recA1 supE44 thi-1 gyrA96 relA1 tonA panD*	Invitrogen, cloning host
XY088	BW25113 *ftsZ*^{E238A}	[6]
XY072	BW25113 *ftsZ*^{E250A}	[6]
XY089	BW25113 *ftsZ*^{D269A}	[6]
XY058	BW25113 *ftsZ*^{G105S}	[6] [same amino acid substitution as *ftsZ84*(Ts)]
DRC14	MC4100 *ftsZ84*(Ts) *leu*::Tn*10*	D. RayChaudhuri (FtsZ amino acid substitution is G105S)
EC251	WT, Weiss lab isolate of MG1655	[9]
EC1532	BL21(DE3) / pDSW730	Transformation, select Amp^{r}
EC1908	EC251 P_{BAD}::*ftsN* (Kan^{r})	[10]
EC5784	BW25113 *ftsZ*^{E250A} *leu*::Tn*10*	P1 DRC14 x XY072, select Tet^{r} at 30°, screen for ability to grow on LB0N at 42°C.
EC5820	*ftsZ*^{G105S} *leu*::Tn*10 ftsB*^{E56A} *ΔftsN*::*kan*	P1 DRC14 x BL173, select Tet^{r} at 30°C, screen Ts on LB0N at 42°C. Confirm *ftsZ* mutation by DNA sequencing.
EC5822	*ftsZ*^{E250A} *leu*::Tn*10 ftsB*^{E56A} *ΔftsN*::*kan*	P1 EC5784 x BL173, select Tet^{r}. Confirm *ftsZ* mutation by DNA sequencing.
JOE565	MC4100 *ftsN*::*kan araD*+	[11]
BL173	TB28 *ftsB*^{E56A} *ΔftsN*::*kan*	[12]
PM6	TB28 *ftsI*^{R167S}	[5]
JXY559	BW25113 *ftsW*^{I302C}	[5]
JM136	TB28 *ftsI(18-19)-Halo*^{SW}	[13]
JL273	EC1908 / pJL098 [$P_{T5\text{-}lac}$::*ftsN* Amp^{r}]	This study
Strains with chromosomal *ftsN* fusions		

EC4240	P_{BAD}::*ftsN* (Kan^{r}) *attP*$_{HK022}$::pDSW1839 [P_{204}::*gfp-ftsN* Spc^{r}]	Integrate pDSW1839 into EC1908 using pAH69
EC4443	P_{BAD}::*ftsN* (Kan^{r}) *attP*$_{\varphi 80}$::pDSW1890 [P_{204_7A}::*mEos3.2-ftsN* Spc^{r}]	Integrate pDSW1890 into EC1908 using pAH123
EC4564	P_{BAD}::*ftsN* (Kan^{r}) *attP*$_{\varphi 80}$::pDSW1926 [P_{204_7A}::*mNG-ftsN* Spc^{r}]	Integrate pDSW1926 into EC1908 using pAH123
EC5230	*attP*$_{\varphi 80}$:: pDSW2083 [P_{204_7A}::*ftsN-Halo*^{E60SW} Spc^{r}]	Integrate pDSW2083 into EC251 using pAH123
EC5234	P_{BAD}::*ftsN* (Kan^{r}) *attP*$_{\varphi 80}$::pDSW2083 [P_{204_7A}::*ftsN-Halo*^{E60SW} Spc^{r}]	Integrate pDSW2083 into EC1908 using pAH123
EC5263	P_{BAD}::*ftsN* (Kan^{r}) *attP*$_{\varphi 80}$::pDSW2099 [P_{204_7A}:: *dsbA*^{ss}-*Halo-ftsN*$^{\Delta Cyto\text{-}TM}$ Spc^{r}]	Integrate pDSW2099 into EC1908 using pAH123
EC5271	P_{BAD}::*ftsN* (Kan^{r}) *attP*$_{\varphi 80}$::pDSW2091 [P_{204_7A}::*ftsN*^{D5N}-*Halo*^{SW} Spc^{r}]	Integrate pDSW2091 into EC1908 using pAH123
EC5317	P_{BAD}::*ftsN* (Kan^{r}) *attP*$_{\varphi 80}$::pDSW2105 [P_{204_7A}::*ftsN*$^{Cyto\text{-}TM}$-*Halo*^{SW} Spc^{r}]	Integrate pDSW2105 into EC1908 using pAH123
EC5321	P_{BAD}::*ftsN* (Kan^{r}) *attP*$_{\varphi 80}$::pDSW2109 [P_{204_7A}::*ftsN*$^{Cyto\text{-}TM\text{-}D5N}$-*Halo*^{SW} Spc^{r}]	Integrate pDSW2109 into EC1908 using pAH123
EC5333	BW25113 *attP*$_{\varphi 80}$::pDSW2083 [P_{204_7A}::*ftsN-Halo*^{E60SW} Spc^{r}]	Integrate pDSW2083 into BW25113 using pAH123
EC5335	BW25113 *ftsZ*^{E238A} *attP*$_{\varphi 80}$::pDSW2083 [P_{204_7A}::*ftsN-Halo*^{E60SW} Spc^{r}]	Integrate pDSW2083 into BW25113 *ftsZ*^{E238A} using pAH123
EC5337	BW25113 *ftsZ*^{E250A} *attP*$_{\varphi 80}$::pDSW2083 [P_{204_7A}::*ftsN-Halo*^{E60SW} Spc^{r}]	Integrate pDSW2083 into BW25113 *ftsZ*^{E250A} using pAH123
EC5339	BW25113 *ftsZ*^{D269A} *attP*$_{\varphi 80}$::pDSW2083 [P_{204_7A}::*ftsN-Halo*^{E60SW} Spc^{r}]	Integrate pDSW2083 into BW25113 *ftsZ*^{D269A} using pAH123
EC5341	BW25113 *ftsZ*^{G105S} *attP*$_{\varphi 80}$::pDSW2083 [P_{204_7A}::*ftsN-Halo*^{E60SW} Spc^{r}]	Integrate pDSW2083 into BW25113 *ftsZ*^{G105S} using pAH123
EC5351	BW25113 P_{BAD}::*ftsN* (Kan^{r}) *attP*$_{\varphi 80}$::pDSW2083 [P_{204_7A}::*ftsN-Halo*^{E60SW} Spc^{r}]	P1 EC1908 x EC5333, select Kan^{r}
EC5353	BW25113 *ftsZ*^{E238A} P_{BAD}::*ftsN* (Kan^{r}) *attP*$_{\varphi 80}$::pDSW2083 [P_{204_7A}::*ftsN-Halo*^{E60SW} Spc^{r}]	P1 EC1908 x EC5335, select Kan^{r}
EC5355	BW25113 *ftsZ*^{E250A} P_{BAD}::*ftsN* (Kan^{r}) *attP*$_{\varphi 80}$::pDSW2083 [P_{204_7A}::*ftsN-Halo*^{E60SW} Spc^{r}]	P1 EC1908 x EC5337, select Kan^{r}
EC5357	BW25113 *ftsZ*^{D269A} P_{BAD}::*ftsN* (Kan^{r}) *attP*$_{\varphi 80}$::pDSW2083 [P_{204_7A}::*ftsN-Halo*^{E60SW} Spc^{r}]	P1 EC1908 x EC5339, select Kan^{r}
EC5359	BW25113 *ftsZ*^{G105S} P_{BAD}::*ftsN* (Kan^{r}) *attP*$_{\varphi 80}$::pDSW2083 [P_{204_7A}::*ftsN-Halo*^{E60SW} Spc^{r}]	P1 EC1908 x EC5341, select Kan^{r}
EC5377	TB28 *ftsI*^{R167S} *attP*$_{\varphi 80}$::pDSW2083 [P_{204_7A}::*ftsN*-Halo^{E60SW} Spc^{r}]	P1 EC5230 x PM6, select Spc^{r}

EC5383	BW25113 *ftsW*^{I302C} *attP*$_{φ80}$::pDSW2083 [P$_{204_7A}$::*ftsN-Halo*^{E60SW} Spc^r]	P1 EC5230 x BW25113 *ftsW*^{I302C}, select Spc^r
EC5387	*ftsI*^{R167S} P$_{BAD}$::*ftsN* (Kan^r) *attP*$_{φ80}$::pDSW2083 [P$_{204_7A}$::*ftsN-Halo*^{E60SW} Spc^r]	P1 EC1908 x EC5377, select Spc^r
EC5391	BW25113 *ftsW*^{I302C} P$_{BAD}$::*ftsN* (Kan^r) *attP*$_{φ80}$::pDSW2083 [P$_{204_7A}$::*ftsN-Halo*^{E60SW} Spc^r]	P1 EC1908 x EC5383, select Kan^r
EC5435	BW25113 *ftsZ*^{E238A} *attP*$_{φ8}$::pDSW2105 [P$_{204_7A}$:: *ftsN*$^{Cyto-TM}$-*Halo*^{SW} Spc^r]	Integrate pDSW2105 into EC5277 using pAH123
EC5437	BW25113 *ftsZ*^{E250A} *attP*$_{φ8}$::pDSW2105 [P$_{204_7A}$:: *ftsN*$^{Cyto-TM}$-*Halo*^{E60SW} Spc^r]	Integrate pDSW2105 into BW25113 *ftsZ*^{E250A} using pAH123
EC5439	BW25113 ftsZ^{D269A} *attP*$_{φ8}$::pDSW2105 [P$_{204_7A}$:: *ftsN*$^{Cyto-TM}$-*Halo*^{E60SW} Spc^r]	Integrate pDSW2105 into BW25113 *ftsZ*^{D269A} using pAH123
EC5441	BW25113 *ftsZ*^{G105S} *attP*$_{φ8}$::pDSW2105 [P$_{204_7A}$:: *ftsN*$^{Cyto-TM}$-*Halo*^{E60SW} Spc^r]	Integrate pDSW2105 into BW25113 *ftsZ*^{G105S} using pAH123
Strains with *ftsN* fusions on plasmids		
EC5606	EC251 P$_{BAD}$::*ftsN* (Kan^r) / pJL113 [P$_{T5-lac}$::*ftsN-Halo*^{SW} Amp^r]	Transform into EC1908, select Amp^r
JL035	JOE565 / pJL019 [P$_{T5-lac}$::*mNG-ftsN* Amp^r]	Transform, select Amp^r
JL080	JOE565 / pJL028 [P$_{T5-lac}$::*ftsN-mNG* Amp^r]	Transform, select Amp^r
JL247	EC1908 / pJL107 [P$_{T5-lac}$::*mNG-ftsN*(P12-A13)-mNG^{SW} Amp^r]	Transform, select Amp^r
JL231	EC1908 / pJL103 [P$_{T5-lac}$::*mNG-ftsN*(N28-L29)-NeG^{SW} Amp^r]	Transform, select Amp^r
JL248	EC1908 / pJL108 [P$_{T5-lac}$::*mNG-ftsN*(E60-E61)-mNG^{SW} Amp^r]	Transform, select Amp^r
JL249	EC1908 / pJL109 [P$_{T5-lac}$::*mNG-ftsN*(K69-V70)-mNG^{SW} Amp^r]	Transform, select Amp^r
JL250	EC1908 / pJL110 [P$_{T5-lac}$::*mNG-ftsN*(Q113-L114)-mNG^{SW} Amp^r]	Transform, select Amp^r
JL251	EC1908 / pJL111 [P$_{T5-lac}$::*mNG-ftsN*(Q124-M125)-mNG^{SW} Amp^r]	Transform, select Amp^r
JL232	EC1908 / pJL100 [P$_{T5-lac}$::*mNG-ftsN*(Q151-T152)-mNG^{SW} Amp^r]	Transform, select Amp^r
JL233	EC1908 / pJL101 [P$_{T5-lac}$::*mNG-ftsN*(Q182-T183)-mNG^{SW} Amp^r]	Transform, select Amp^r
JL234	EC1908 / pJL102 [P$_{T5-lac}$::*mNG-ftsN*(Q212-T213)-mNG^{SW} Amp^r]	Transform, select Amp^r
JL364	EC5377 / pCH650 [pACYC*, cat araC* P$_{BAD}$::*uppS*]	Transform, select Cam^r
JL397	BL173 / pJL132 [P$_{T5-lac}$::*ftsN-Halo*^{SW} Cam^r]	Transform, select Cam^r

JL398	BL173 / pJL133 [$P_{T5\text{-}lac}$::*ftsN*^{WYAA}-*Halo*^{SW} Cam^r]	Transform, select Cam^r
JL399	BL173 / pJL136 [$P_{T5\text{-}lac}$:: *dsbA*^{ss}-*Halo*-*ftsN*$^{61\text{-}105}$ Cam^r]	Transform, select Cam^r
JL421	EC5820 / pJL136 [$P_{T5\text{-}lac}$:: *dsbA*^{ss}-*Halo*-*ftsN*$^{61\text{-}105}$ Cam^r]	Transform, select Cam^r
JL422	EC5822 / pJL136 [$P_{T5\text{-}lac}$:: *dsbA*^{ss}-*Halo*-*ftsN*$^{61\text{-}105}$ Cam^r]	Transform, select Cam^r

Supplementary Table 2. Plasmids used in this study.

Plasmid	Relevant features/description	Source or reference
pAH69	λcI*857*, *rep101*^{ts} ori, P_r-*int*$_{HK022}$, Amp^r	[14]
pAH123	λcI*857*, *rep101*^{ts} ori, P_r-*int*$_{\Phi 80}$, Amp^r	[14]
pDSW254	P_{204}::*gfp-ftsI lacI*^{Q} Kan^r pBR ori	[15]
pDSW238	P_{204}::*gfp-ftsN* Amp^r pBR ori	[16]
pDSW499	Promoterless *gfp*-MCS *oriR*$_\gamma$ *attP*$_{HK022}$ Spc^r	[17]
pDSW534	P_{204}::*gfp*-MCS lacI^Q *oriR*$_\gamma$ *attP*$_{HK022}$ Spc^r	This study
pDSW984	P_{204}::*gfp-dedD lacI*^{Q} *oriR*$_\gamma$ *attP*$_{\Phi 80}$ Spc^r	[18]
pDSW730	$P_{T5/lac}$::*His*$_6$*-ftsN*^{peri} lacI^Q Amp^r pBR ori	This study
pDSW1198	P_{204}::*gfp lacI*^{Q} *oriR*$_\gamma$ *attP*$_{\Phi 80}$ Spc^r	[19]
pDSW1839	P_{204}::*gfp-ftsN lacI*^{Q} *oriR*$_\gamma$ *attP*$_{HK022}$ Spc^r	This study
pDSW1866	P_{204_7A}::*gfp-dedD lacI*^{Q} *oriR*$_\gamma$ *attP*$_{\Phi 80}$ Spc^r	This study
pDSW1876	P_{204_7A}::*gfp-BamHI lacI*^{Q*} *oriR*$_\gamma$ *attP*$_{\Phi 80}$ Spc^r	This study
pDSW1884	P_{204_7A}::*mEos3.2-MCS lacI*^{Q} *oriR*$_\gamma$ *attP*$_{\Phi 80}$ Spc^r	This study
pDSW1890	P_{204_7A}::*mEos3.2-ftsN lacI*^{Q} *oriR*$_\gamma$ *attP*$_{\Phi 80}$ Spc^r	This study
pDSW1920	P_{204_7A}::*Halo-ftsN lacI*^{Q} *oriR*$_\gamma$ *attP*$_{\Phi 80}$ Spc^r	This study
pDSW1926	P_{204_7A}::*mNG-ftsN lacI*^{Q} *oriR*$_\gamma$ *attP*$_{\Phi 80}$ Spc^r	This study
pDSW2031	P_{204_7A}::*ftsN-mNG*^{E60SW} *lacI*^{Q} *oriR*$_\gamma$ *attP*$_{\Phi 80}$ Spc^r	This study
pDSW2035	P_{204_7A}::*ftsN-Halo*^{Q141SW} *lacI*^{Q*} *oriR*$_\gamma$ *attP*$_{\Phi 80}$ Spc^r	This study
pDSW2083	P_{204_7A}::*ftsN-Halo*^{E60SW} *lacI*^{Q*} *oriR*$_\gamma$ *attP*$_{\Phi 80}$ Spc^r	This study
pDSW2091	P_{204_7A}::*ftsN*^{D5N}*-Halo*^{E60SW} *lacI*^{Q*} *oriR*$_\gamma$ *attP*$_{\Phi 80}$ Spc^r	This study
pDSW2099	P_{204_7A}:: *dsbA*^{ss}*-Halo-ftsN*$^{\Delta cyto-TM}$ *lacI*^{Q*} *oriR*$_\gamma$ *attP*$_{\Phi 80}$ Spc^r	This study
pDSW2105	P_{204_7A}::*ftsN*$^{Cyto-TM}$*-Halo*^{E60SW} *lacI*^{Q*} *oriR*$_\gamma$ *attP*$_{\Phi 80}$ Spc^r	This study
pDSW2109	P_{204_7A}::*ftsN*$^{Cyto-TM-D5N}$*-Halo*^{E60SW} *lacI*^{Q*} *oriR*$_\gamma$ *attP*$_{\Phi 80}$ Spc^r	This study
pXY027	ColE1, P_{T5-lac}::*ftsZ-GFP*, Cam^r	[20]
pXY677	ColE1, P_{T5-lac}::*mNG-zapA*, Cam^r	[5]
pJL015	ColE1, P_{T5-lac}::*mEos3.2-ftsN*, Amp^r	[4]
pJL028	ColE1, P_{T5-lac}::*ftsN-mNG*, Amp^r	This study
pJL033	ColE1, P_{T5-lac}::*Halo-ftsN*, Amp^r	This study
pJL069	P_{204_7A}:: *Halo-ftsN*$^{243-319}$ *lacI*^{Q*} *oriR*$_\gamma$ *attP*$_{\Phi 80}$ Spc^r	This study
pJL074	P_{204_7A}:: *dsbA*^{ss}*-Halo-ftsN*$^{243-319}$ *lacI*^{Q*} *oriR*$_\gamma$ *attP*$_{\Phi 80}$ Spc^r	This study
pJL098	ColE1, P_{T5-lac}::*ftsN*, Amp^r	This study
pJL019	ColE1, P_{T5-lac}::*mNG-ftsN*, Amp^r	This study
pJL107	ColE1, P_{T5-lac}::*ftsN(P12-A13)-mNG*^{SW}, Amp^r	This study
pJL103	ColE1, P_{T5-lac}::*ftsN(N28-L29)-mNG*^{SW}, Amp^r	This study
pJL108	ColE1, P_{T5-lac}::*ftsN(E60-E61)-mNG*^{SW}, Amp^r	This study
pJL109	ColE1, P_{T5-lac}::*ftsN(K69-V70)-mNG*^{SW}, Amp^r	This study
pJL110	ColE1, P_{T5-lac}::*ftsN(Q113-L114)-mNG*^{SW}, Amp^r	This study
pJL111	ColE1, P_{T5-lac}::*ftsN(Q124-M125)-mNG*^{SW}, Amp^r	This study
pJL100	ColE1, P_{T5-lac}::*ftsN(Q151-T152)-mNG*^{SW}, Amp^r	This study
pJL101	ColE1, P_{T5-lac}::*ftsN(Q182-T183)-mNG*^{SW}, Amp^r	This study

pJL102	ColE1, $P_{T5\text{-}lac}$::*ftsN(Q212-T213)-mNG*^{SW}, Amp^{r}	This study
pJL112	ColE1, $P_{T5\text{-}lac}$::*ftsN(Q151-T152)-Halo*^{SW}, Amp^{r}	This study
pJL113	ColE1, $P_{T5\text{-}lac}$::*ftsN(E60-E61)-Halo*^{SW}, Amp^{r}	This study
pJL119	ColE1, $P_{T5\text{-}lac}$::*ftsN*, Cam^{r}	This study
pJL123	ColE1, $P_{T5\text{-}lac}$::*dsbA*^{ss}-*ftsN*$^{243\text{-}319}$, Cam^{r}	This study
pJL132	ColE1, $P_{T5\text{-}lac}$::*ftsN(E60-E61)-Halo*^{SW}, Cam^{r}	This study
pJL133	ColE1, $P_{T5\text{-}lac}$::*ftsN(E60-E61, WYAA)-Halo*^{SW}, Cam^{r}	This study
pJL135	ColE1, $P_{T5\text{-}lac}$::*dsbA*^{ss}-*ftsN*$^{61\text{-}105}$, Cam^{r}	This study
pJL136	ColE1, $P_{T5\text{-}lac}$::*dsbA*^{ss}-*Halo-ftsN*$^{61\text{-}105}$, Cam^{r}	This study
pCH650	pACYC, *cat araC* P_{BAD}::*uppS*	[5]

pDSW534. A 1706 bp fragment encoding *lacI*^{Q} and P_{204}::*gfp* was obtained by digesting pDSW254 with NdeI, SphI and XmnI. The fragment was ligated into pDSW499 after digestion with NdeI and SphI.

pDSW730. Amplify periplasmic domain of *ftsN* with primers P760 and P761. The 833 bp fragment was digested with BamHi and HindIII, then ligated into the same sites of pQE-80L (Qiagen).

pDSW1839. Amplify *gfp-ftsN* from pDSW238 with primers P2108 and P2109. The 1142 bp product was digested with MfeI and SacI, then ligated into the same sites of pDSW534.

pDSW1866. The P_{204} promoter in pDSW984 was replaced with a weaker P_{204_7A} promoter using isothermal assembly to insert a 675 bp gBlock into the SfoI and NdeI sites of pDSW984.

pDSW1876. The P_{204} promoter in pDSW1198 was replaced with a weaker P_{204_7A} promoter using isothermal assembly to insert a 675 bp gBlock into the SfoI and NdeI sites of pDSW1198. The *lacI*^{Q} allele is designated *lacI*^{Q*} because it was later found to have a frame shift mutation, a deletion of T999. The last 28 amino acids of wild-type LacI (NTQTASPRALADSLMQLARQVSRLESGQ) become KRKPPLPARWPIH. The mutant Lac repressor is still active but does not repress as well as wild-type, so leaky expression is about two-fold higher.

pDSW1884. Amplify mEos3.2 from pJL015 with primers P2163 and P2164. The 757 bp fragment was digested with AflII and MfeI, then ligated into AflII-EcoRI digested pDSW1866.

pDSW1890. Amplify *ftsN* from pJL015 with P2178 and P2179. The 1029 bp product was digested with EcoRI and BamHI, and ligated into the same sites of pDSW1884.

pDSW1920. Amplify *Halo* from pJL033 with primers P2228 and P2225. The 927 bp product was digested with AflII and EcoRI, then ligated into the same sites of pDS1890.

pDSW1926. Amplify mNeonGreen from pJL019 with primers P2222 and P2223. The 744 bp product was digested with AflII and EcoRI, then ligated into the same sites of pDS1890.

pDSW2031. Amplify the *ftsN*(E60-mNG) sandwich fusion from pJL108 with P2392 and P2393. The 1730 bp product was digested with AflII and BamHI, then ligated into the same sites of pDSW1876.

pDSW2035. Amplify the *ftsN*(Q151-Halo) sandwich fusion from pJL112 with P2392 and P2394. The 1916 bp product was digested with AflII and BamHI, then ligated into the same sites of pDSW1876.

pDSW2083. Constructed from a four-fragment Gibson Assembly. The vector backbone was obtained by digestion of pDSW2035 with AflII and KpnI. The inserts were a 231 bp fragment amplified from pDSW2031 with primers P2439 and P2445, an 891 bp fragment amplified from pDSW2035 with primers P2446 and P2447, and an 804 bp fragment amplified from pDSW2031 with primers P2448 and P2449.

pDSW2091. Amplify the *ftsN* (E60-Halo) sandwich fusion from pDSW2083 using primers P2422 and P2460. P2422 introduces a D5N amino acid substitution. The 2009 bp product was digested with AflII and KpnI, then ligated into the same sites of pDSW2035.

pDSW2099. Obtain a 946 bp $dsbA^{ss}$-Halo fragment from pJL074 by digestion with AflII and AccI. This fragment was ligated into the same sites of pDSW2083.

pDSW2105. Amplify a fragment of the *ftsN*(E60-Halo) sandwich fusion from pDSW2083 with primers P2182 and P2525. The 1357 bp PCR product encodes *ftsN* residues 1-73, a Halo tag inserted between *ftsN* residues 60-61, followed by *ftsN* residues 61-73. This DNA fragment was digested with AflII and KpnI, then ligated into the same sites of pDSW2035.

pDSW2109. Amplify part of the $ftsN^{D5N}$ (E60-Halo) sandwich fusion from pDSW2091 using primers P2182 and P2525. The 1357 bp product was digested with AflII and KpnI, then ligated into the same sites of pDSW2035.

pJL033. Amplify *Halo* gene from the chromosome of strain JM136 which contains the sandwich *Halo-ftsI* gene[13] with primers 19 and 20. Amplify *ftsN* gene with the vector backbone from pJL015 with primers 13 and 72. The two DNA fragments were then joined by the In-Fusion Cloning Kit to generate plasmid pJL033 ($P_{T5\text{-}lac}$::*Halo-ftsN*, Amp^r).

pJL069. Amplify *Halo* gene with the vector backbone from pDSW1920 (P_{204_7A}:: *Halo-ftsN* $lacI^{Q}$* $oriR_{\gamma}$ $attP_{\Phi 80}$ Spc^r) with primers 89 and 90. Amplify $ftsN^{243\text{-}319}$ gene from pJL028 with primers 66 and 92. The two DNA fragments were then joined by the In-Fusion Cloning Kit to generate plasmid pJL069 (P_{204_7A}:: $Halo\text{-}ftsN^{243\text{-}319}$ $lacI^{Q}$* $oriR_{\gamma}$ $attP_{\Phi 80}$ Spc^r).

pJL074. The pJL074 (P_{204_7A}:: $dsbA^{ss}$-$Halo\text{-}ftsN^{243\text{-}319}$ $lacI^{Q}$* $oriR_{\gamma}$ $attP_{\Phi 80}$ Spc^r) plasmid was constructed from the pJL069 plasmid using the QuikChange protocol (Agilent) with the primers 111 and 112 to insert the $dsbA^{ss}$ gene in front of *Halo* gene.

pJL098. Amplify the vector backbone from pJL015 ($P_{T5\text{-}lac}$::*mEos3.2-ftsN*) [4] with primers 13 and 72. Amplify *ftsN* gene from pJL015 with primers 127 and 128. The two DNA fragments were then joined by the In-Fusion Cloning Kit to generate plasmid pJL098 ($P_{T5\text{-}lac}$::*ftsN*, Amp^r).

pJL019. Amplify *ftsN* gene with the vector backbone from pJL015 with primers 13 and 72. Amplify *mNeonGreen* gene from pXY677 ($P_{T5\text{-}lac}$::*mNeonGreen-zapA*)[5] with primers 11 and 12. The two DNA fragments were then joined by the In-Fusion Cloning Kit to generate plasmid pJL019 ($P_{T5\text{-}lac}$::*mNG-ftsN*).

pJL107. Amplify *ftsN*$^{12\text{-}13}$ gene with the vector backbone from pJL098 with primers 148 and149. Amplify *mNeonGreen* gene from pJL019 with primers 150 and 151. The two DNA fragments were then joined by the In-Fusion Cloning Kit to generate plasmid pJL107 ($P_{T5\text{-}lac}$::*ftsN(P12-A13)-mNG*^{SW}).

pJL103. Amplify *ftsN*$^{28\text{-}29}$ gene with the vector backbone from pJL098 with primers 141 and142. Amplify *mNeonGreen* gene from pJL019 with primers 143 and 144. The two DNA fragments were then joined by the In-Fusion Cloning Kit to generate plasmid pJL103 ($P_{T5\text{-}lac}$::*ftsN(N28-L29)-mNG*^{SW}).

pJL108. Amplify *ftsN*$^{60\text{-}61}$ gene with the vector backbone from pJL098 with primers 152 and153. Amplify *mNeonGreen* gene from pJL019 with primers 154 and 155. The two DNA fragments were then joined by the In-Fusion Cloning Kit to generate plasmid pJL108 ($P_{T5\text{-}lac}$::*ftsN(E60-E61)-mNG*^{SW}).

pJL109. Amplify *ftsN*$^{69\text{-}70}$ gene with the vector backbone from pJL098 with primers 156 and157. Amplify *mNeonGreen* gene from pJL019 with primers 158 and 159. The two DNA fragments were then joined by the In-Fusion Cloning Kit to generate plasmid pJL109 ($P_{T5\text{-}lac}$::*ftsN(K69-V70)-mNG*^{SW}).

pJL110. Amplify *ftsN*$^{113\text{-}114}$ gene with the vector backbone from pJL098 with primers 160 and161. Amplify *mNeonGreen* gene from pJL019 with primers 162 and 163. The two DNA fragments were then joined by the In-Fusion Cloning Kit to generate plasmid pJL110 ($P_{T5\text{-}lac}$::*ftsN(Q113-L114)-mNG*^{SW}).

pJL111. Amplify *ftsN*$^{151\text{-}152}$ gene with the vector backbone from pJL098 with primers 164 and165. Amplify *mNeonGreen* gene from pJL019 with primers 166 and 167. The two DNA fragments were then joined by the In-Fusion Cloning Kit to generate plasmid pJL111 ($P_{T5\text{-}lac}$::*ftsN(Q124-M125)-mNG*^{SW}).

pJL100. Amplify *ftsN*$^{124\text{-}125}$ gene with the vector backbone from pJL098 with primers 129 and130. Amplify *mNeonGreen* gene from pJL019 with primers 131 and 132. The two DNA fragments were then joined by the In-Fusion Cloning Kit to generate plasmid pJL100 ($P_{T5\text{-}lac}$::*ftsN(Q151-T152)-mNG*^{SW}).

pJL101. Amplify *ftsN*$^{182\text{-}183}$ gene with the vector backbone from pJL098 with primers 133 and134. Amplify *mNeonGreen* gene from pJL019 with primers 135 and 136. The two DNA

fragments were then joined by the In-Fusion Cloning Kit to generate plasmid pJL101 ($P_{T5\text{-}lac}$::*ftsN(Q182-T183)-mNG*^{SW}).

pJL102. Amplify *ftsN*$^{212\text{-}213}$ gene with the vector backbone from pJL098 with primers 137 and138. Amplify *mNeonGreen* gene from pJL019 with primers 139 and 140. The two DNA fragments were then joined by the In-Fusion Cloning Kit to generate plasmid pJL102 ($P_{T5\text{-}lac}$::*ftsN(Q212-T213)-mNG*^{SW}).

pJL028. Amplify *ftsN* gene with the vector backbone from pJL015 with primers 13 and 72. Amplify *mNeonGreen* gene from pJL019 with primers 39 and 40. The two DNA fragments were then joined by the In-Fusion Cloning Kit to generate plasmid pJL028 ($P_{T5\text{-}lac}$::*ftsN-mNG*).

pJL112. Amplify *ftsN*$^{151\text{-}152}$ gene with the vector backbone from pJL098 with primers 170 and171. Amplify *Halo* gene from pJL033 with primers 172 and 173. The two DNA fragments were then joined by the In-Fusion Cloning Kit to generate plasmid pJL113 ($P_{T5\text{-}lac}$::*ftsN(Q151-T152)-Halo*^{SW}, Amp^r).

pJL113. Amplify *ftsN*$^{60\text{-}61}$ gene with the vector backbone from pJL098 with primers 152 and153. Amplify *Halo* gene from pJL033 with primers 177 and 178. The two DNA fragments were then joined by the In-Fusion Cloning Kit to generate plasmid pJL113 ($P_{T5\text{-}lac}$::*ftsN(E60-E61)-Halo*^{SW}, Amp^r).

pJL119. Amplify the vector backbone from pXY027 ($P_{T5\text{-}lac}$::*ftsZ-GFP*, Cam^r)[20] with primers 72 and 126. Amplify *ftsN* gene from pJL015 with primers 127 and 128. The two DNA fragments were then joined by the In-Fusion Cloning Kit to generate plasmid pJL119 ($P_{T5\text{-}lac}$::*ftsN*, Cam^r).

pJL123. Amplify *ftsN*$^{243\text{-}319}$ gene with the vector backbone from pJL119 with primers 72 and 106. Amplify *dsbA*^{ss} gene from pJL074 with primers 181 and 183. The two DNA fragments were then joined by the In-Fusion Cloning Kit to generate plasmid pJL123 ($P_{T5\text{-}lac}$::*dsbA*^{ss}-*ftsN*$^{243\text{-}319}$, Cam^r).

pJL132. Amplify *ftsN*$^{60\text{-}61}$ gene with the vector backbone from pJL119 with primers 152 and153. Amplify *Halo* gene from pJL113 with primers 177 and 178. The two DNA fragments were then joined by the In-Fusion Cloning Kit to generate plasmid pJL132 ($P_{T5\text{-}lac}$::*ftsN(E60-E61)-Halo*^{SW}, Cam^r).

pJL133. The pJL133 ($P_{T5\text{-}lac}$::*ftsN(E60-E61, WYAA)-Halo*^{SW}, Cam^r) plasmid was constructed from the pJL132 plasmid using the QuikChange protocol (Agilent) with the primers 60 and 61 to mutate the nucleotide sequence encoding for W83A, Y85A.

pJL135. Amplify *dsbA*^{ss} gene with the vector backbone from pJL123 with primers 116 and 126. Amplify *ftsN*$^{61\text{-}105}$ gene from pJL119 with primers 184 and 187. The two DNA fragments were then joined by the In-Fusion Cloning Kit to generate plasmid pJL135 ($P_{T5\text{-}lac}$::*dsbA*^{ss}-*ftsN*$^{61\text{-}105}$, Cam^r).

pJL136. Amplify *dsbA*^{ss} gene and *ftsN*$^{61\text{-}105}$ gene with the vector backbone from pJL123 with primers 116 and 152. Amplify *Halo* gene from pJL113 with primers 76 and 178. The two DNA fragments were then joined by the In-Fusion Cloning Kit to generate plasmid pJL136 ($P_{T5\text{-}lac}$::*dsbA*^{ss}-*Halo*-*ftsN*$^{61\text{-}105}$, Cam^{r}).

Supplementary Table 3. Oligonucleotides used in this study.

Name	Sequence (5'->3')	Comment
P760	CGGGATCCGGTGGTCTGTACTTCATTACG	Forward primer to clone *ftsN* periplasmic domain into pQE-80L
P761	CCCAAGCTTTCAACCCCCGGCGGCGAGCCG	Reverse primer to clone *ftsN* periplasmic domain into pQE-80L
P2108	CTCCAATTGGCGATGGCCCTGTCCT	Forward primer to clone *gfp-ftsN* into pDSW534
P2109	GATGAGCTCTCAACCCCCGGCGGCGAG	Reverse primer to clone *gfp-ftsN* into pDSW534
P2163	CAGCTTAAGACACAGGAAACAGACCATGAGTGCGATTAAGCCAG	Forward primer to clone *mEos3.2* into pDSW1866
P2164	CTGCAATTGCTGCAGGTCGACTCTAGAGGATCCCCGGGTACCGAGCTCGAATTCTCGTCTGGCATTGTCAGG	Reverse primer to clone *mEos3.2* into pDSW1866
P2178	CCAGAATTCATCAACAAGTTTGTACAAAAAAGCAGGCTC	Forward primer to clone *ftsN* into pDSW1884
P2179	TGGGGATCCTCAACCCCCGGCGGCGAG	Reverse primer to clone *ftsN* into pDSW1884
P2182	CTGTCTACTCTGGAGATTTCCGGTGGTGGCGGTGGTAGTGCGGAGAAAAAAGACGAACGC	Forward primer to clone *ftsN*$^{\text{Cyto-TM}}$ *and ftsN*$^{\text{D5N-Cyto-TM}}$ into pDSW2035
P2222	CAATTCTTAAGACACAGGAAACAGACCATGGTGAGCAAAGGCGAAGAAG	Forward primer to clone *mNeonGreen* into pDSW1890
P2223	GTGGAATTCTTTATACAGTTCATCCATGCCCATC	Reverse primer to clone *mNeonGreen* into pDSW1890
P2225	GTGGAATTCACCGGAAATCTCCAGAGTAG	Reverse primer to clone *halo* into pDSW1890
P2228	CAATTCTTAAGACACAGGAAACAGACCATGGCAGAAATCGGTACTGGCTTTCCATTC	Forward primer to clone *halo* into pDSW1890
P2392	CACCTTAAGACACAGGAAACAGACC ATGGCACAACGAGATTATGTAC	Forward primer to clone *ftsN* sandwich fusions into pDSW1876
P2393	CACGGGATCCTTAACCCCCGGCGG	Reverse primer to clone *ftsN* sandwich fusions into pDSW1876
P2394	CACGAGATCTTTAACCCCCGGCGG	Alternative reverse primer to clone sandwich fusions into pDSW1876
P2422	CAATTCTTAAGACACAGGAAACAGACCATGGCACAACGAAATTATGTACGCCG	Forward primer to clone *ftsN*$^{\text{D5N}}$-*Halo*^{SW} into pDSW2035; introduces D5N substitution

P2439	GAGCGGATAACAATTCTTAAGACACAGGAAACAGAC CATGGC	Forward primer for constructing *ftsN-Halo*^{SW} plasmid
P2445	TTTCGGATCCGCTACCACCGCCACCTTC	Reverse primer for constructing *ftsN-Halo*^{SW} plasmid
P2446	CGGTGGTAGCGGATCCGAAATCGGTACTGGCT	Forward primer for constructing *ftsN-Halo*^{SW} plasmid
P2447	CACCGCCACCACCGGAAATCTCCAGAGTAGACAGC	Reverse primer for constructing *ftsN-Halo*^{SW} plasmid
P2448	GATTTCCGGTGGTGGCGGTGGTAGCGAGTCC	Forward primer for constructing *ftsN-Halo*^{SW} plasmid
P2449	AACATGAGAATTCGAGCTCGGTACCCGGGGATCCTT AACCCCCG	Reverse primer for constructing *ftsN-Halo*^{SW} plasmid
P2460	CTAGAGGATCCCGTGGAAAAATGTGACTTTTATCAC	Forward primer to clone *ftsN*^{D5N}-*Halo*^{SW} into pDSW2035
P2525	CTCGGTACCCGGGGATCCTTAGTTTCCGGTCACTTT CTGGCTTTG	Reverse primer to clone *ftsN*$^{Cyto-TM}$ *and ftsN*$^{D5N-Cyto-TM}$ into pDSW2035
11	GGAGAAATTAACTACTAGTATGGTGAGCAAGGCGA AGAAG	Forward primer to clone *mNG* into pJL019
12	GCTTTTTTGTACAAACTTGTTGATTTTATACAGTTCAT CCATGCCCATC	Reverse primer to clone *mNG* into pJL019
13	ATCAACAAGTTTGTACAAAAAAGCAGG	Forward primer to amplify *ftsN*-vector fragment for pJL019 and pJL033
19	GAGGAGAAATTAACTACTAGTATGGGATCCGAAATC GGTACTG	Forward primer to clone *halo* into pJL033
20	GAGCCTGCTTTTTTGTACAAACTTGTTGATACCGGA AATCTCCAGAGTAGAC	Reverse primer to clone *halo* into pJL033
39	AAAAGCAGGCTCCGCGGCCGCCCCCTTCACCAAGT GAGCAAAGGCGAAGAAGATAAC	Forward primer to clone *mNG* into pJL028
40	GGCTGCAGGTCGACCCTTAGCGGCCGCTTATTTATA CAGTTCATCCATGCCCATC	Reverse primer to clone *mNG* into pJL028
60	CCAGAAGAACGCGCTCGCGCCATTAAAGAGCTG	Forward primer for site-specific mutagenesis into WYAA for pJL133
61	CAGCTCTTTAATGGCGCGAGCGCGTTCTTCTGG	Reverse primer for site-specific mutagenesis into WYAA for pJL133
66	CCGCCCCCTTCACCAAAAAGACGAACGCCGCTGGA TGG	Forward primer to clone *ftsN*$^{243-319}$ into pJL069
72	ACTAGTAGTTAATTTCTCCTCTTTAATG	Reverse primer to amplify vector fragment

		for pJL019, pJL033, pJL109 and pJL123
76	CTGGCTGGTTTAGTTTTAGCGTTTAGCGCATCGGCG GGATCCGAAATCGGTACTGG	Forward primer to clone *halo* into pJL136
89	TTGGTGAAGGGGGCGGCC	Forward primer to amplify *halo*-vector fragment for pJL069
90	GGATCCTCTAGAGTCGACCT	Reverse primer to amplify *Halo*-vector fragment for pJL069
92	GGTCGACTCTAGAGGATCCTCAACCCCCGGCGGCG	Reverse primer to clone *ftsN*$^{243\text{-}319}$ into pJL069
106	AAAGACGAACGCCGCTGGATGG	Forward primer to amplify *ftsN*$^{243\text{-}319}$-vector fragment for pJL123
111	CTGGCTGGTTTAGTTTTAGCGTTTAGCGCATCGGCG GCAGAAATCGGTACTGGCTTTC	Forward primer to clone *dsbA*^{ss} into pJL074
112	GCTAAAACTAAACCAGCCAGCGCCAGCCAAATCTTT TTCATGGTCTGTTTCCTGTGTCTTAAGAA	Reverse primer to clone *dsbA*^{ss} into pJL074
116	CGCCGATGCGCTAAACGCT	Reverse primer to amplify *dsbA*^{ss}-vector fragment for pJL135 and pJL136
126	GCGGCCGCTAAGGGTCG	Forward primer to amplify vector fragment for pJL119 and pJL135
127	GAGGAGAAATTAACTACTAGTATGGCACAACGAGAT TATGTACGC	Forward primer to clone *ftsN* into pJL098 and pJL119
128	GACCCTTAGCGGCCGCTTAACCCCCGGCGGCGAGC	Reverse primer to clone *ftsN* into pJL098 and pJL119
129	GCTACCACCGCCACCTTGCTGACGCTGTTCCGGC	Forward primer to amplify *ftsN*$^{151\text{-}152}$-vector fragment for pJL100
130	GGTGGCGGTGGTAGCACGCTACAGCGCCAACGTC	Reverse primer to amplify *ftsN*$^{151\text{-}152}$-vector fragment for pJL100
131	GCGTCAGCAAGGTGGCGGTGGTAGCGTGAGCAAAG GCGAAGAAGATAAC	Forward primer to clone *mNG* into pJL100
132	GTAGCGTGCTACCACCGCCACCTTTATACAGTTCAT CCATGCCCATC	Reverse primer to clone *mNG* into pJL100
133	GCTACCACCGCCACCCTGCTGCTGCCAGCTTTGTTC	Forward primer to amplify *ftsN*$^{182\text{-}183}$-vector fragment for pJL101
134	GGTGGCGGTGGTAGCACGCGTACGTCGCAAGCCG	Reverse primer to amplify *ftsN*$^{182\text{-}183}$-vector fragment for pJL101
135	CAGCAGCAGGGTGGCGGTGGTAGCGTGAGCAAAG GCGAAGAAGATAAC	Forward primer to clone *mNG* into pJL101

136	CGTACGCGTGCTACCACCGCCACCTTTATACAGTTCATCCATGCCCATC	Reverse primer to clone *mNG* into pJL101
137	GCTACCACCGCCACCTTGCAGCAGATCCTGGTACGG	Forward primer to amplify *ftsN*$^{212-213}$-vector fragment for pJL102
138	GGTGGCGGTGGTAGCACTCCTGCGCACACGACTGC	Reverse primer to amplify *ftsN*$^{212-213}$-vector fragment for pJL102
139	CTGCAAGGTGGCGGTGGTAGCGTGAGCAAAGGCGAAGAAGATAAC	Forward primer to clone *mNG* into pJL102
140	CGCAGGAGTGCTACCACCGCCACCTTTATACAGTTCATCCATGCCCATC	Reverse primer to clone *mNG* into pJL102
141	GCTACCACCGCCACCATTTCGTTGCTTTTTCCGTGAGGTG	Forward primer to amplify *ftsN*$^{28-29}$-vector fragment for pJL103
142	GGTGGCGGTGGTAGCCTGCCTGCGGTTTCTCCCG	Reverse primer to amplify *ftsN*$^{28-29}$-vector fragment for pJL103
143	GCAACGAAATGGTGGCGGTGGTAGCGTGAGCAAAGGCGAAGAAGATAAC	Forward primer to clone *mNG* into pJL103
144	GCAGGCAGGCTACCACCGCCACCTTTATACAGTTCATCCATGCCCATC	Reverse primer to clone *mNG* into pJL103
148	GGTGGCGGTGGTAGCGCACCTTCGCGGCGAAAAAAG	Forward primer to amplify *ftsN*$^{12-13}$-vector fragment for pJL107
149	GCTACCACCGCCACCCGGTTGGCTGCGGCGTACA	Reverse primer to amplify *ftsN*$^{12-13}$-vector fragment for pJL107
150	CCAACCGGGTGGCGGTGGTAGCGTGAGCAAAGGCGAAGAAGATAAC	Forward primer to clone *mNG* into pJL107
151	CGAAGGTGCGCTACCACCGCCACCTTTATACAGTTCATCCATGCCCATC	Reverse primer to clone *mNG* into pJL107
152	GGTGGCGGTGGTAGCGAGTCCGAGACGCTGCAAAG	Forward primer to amplify *ftsN*$^{60-61}$-vector fragment for pJL108 and pJL136
153	GCTACCACCGCCACCTTCTTTCTTGTGATGCGTAATGAAGTAC	Reverse primer to amplify *ftsN*$^{60-61}$-vector fragment for pJL108
154	CAAGAAAGAAGGTGGCGGTGGTAGCGTGAGCAAAGGCGAAGAAGATAAC	Forward primer to clone *mNG* into pJL108
155	CGGACTCGCTACCACCGCCACCTTTATACAGTTCATCCATGCCCATC	Reverse primer to clone *mNG* into pJL108
156	GGTGGCGGTGGTAGCGTGACCGGAAACGGACTACC	Forward primer to amplify *ftsN*$^{69-70}$-vector fragment for pJL109
157	GCTACCACCGCCACCTTTCTGGCTTTGCAGCGTCTCG	Reverse primer to amplify *ftsN*$^{69-70}$-vector fragment for pJL109

158	CCAGAAAGGTGGCGGTGGTAGCGTGAGCAAAGGCGAAGAAGATAAC	Forward primer to clone *mNG* into pJL109
159	GGTCACGCTACCACCGCCACCTTTATACAGTTCATCCATGCCCATC	Reverse primer to clone *mNG* into pJL109
160	GGTGGCGGTGGTAGCCTGACACCAGAACAACGTCAGC	Forward primer to amplify *ftsN*$^{113-114}$-vector fragment for pJL110
161	GCTACCACCGCCACCTTGCTCCGGCGTTTTCACTTCAC	Reverse primer to amplify *ftsN*$^{113-114}$-vector fragment for pJL110
162	CGGAGCAAGGTGGCGGTGGTAGCGTGAGCAAAGGCGAAGAAGATAAC	Forward primer to clone *mNG* into pJL110
163	GTGTCAGGCTACCACCGCCACCTTTATACAGTTCATCCATGCCCATC	Reverse primer to clone *mNG* into pJL110
164	GGTGGCGGTGGTAGCATGCAGGCTGATATGCGCCAG	Forward primer to amplify *ftsN*$^{124-125}$-vector fragment for pJL111
165	GCTACCACCGCCACCTTGTTCAAGAAGCTGACGTTGTTCTG	Reverse primer to amplify *ftsN*$^{124-125}$-vector fragment for pJL111
166	GAACAAGGTGGCGGTGGTAGCGTGAGCAAAGGCGAAGAAGATAAC	Forward primer to clone *mNG* into pJL111
167	CCTGCATGCTACCACCGCCACCTTTATACAGTTCATCCATGCCCATC	Reverse primer to clone *mNG* into pJL111
170	GCTACCACCGCCACCTTGC	Forward primer to amplify *ftsN*$^{151-152}$-vector fragment for pJL112
171	GGTGGCGGTGGTAGCACG	Reverse primer to amplify *ftsN*$^{151-152}$-vector fragment for pJL112
172	GCGTCAGCAAGGTGGCGGTGGTAGCGGATCCGAAATCGGTACTGGC	Forward primer to clone *halo* into pJL112
173	GCTGTAGCGTGCTACCACCGCCACCACCGGAAATCTCCAGAGTAGAC	Reverse primer to clone *halo* into pJL112
177	CAAGAAAGAAGGTGGCGGTGGTAGCGGATCCGAAATCGGTACTGGC	Forward primer to clone *Halo* into pJL132
178	CTCGGACTCGCTACCACCGCCACCACCGGAAATCTCCAGAGTAGAC	Reverse primer to clone *Halo* into pJL132 and pJL136
181	CATTAAAGAGGAGAAATTAACTACTAGTATGAAAAAGATTTGGCTGGCGCTG	Forward to clone *dsbA*^{ss} into pJL123
183	CTGCACCATCCAGCGGCGTTCGTCTTTCGCCGATGCGCTAAACGCTAA	Reverse primer to clone *dsbA*^{ss} into pJL123
184	GTTTAGTTTTAGCGTTTAGCGCATCGGCGGAGTCCGAGACGCTGCAAAGC	Forward primer to clone *ftsN*$^{61-105}$ into pJL135
187	CTGCAGGTCGACCCTTAGCGGCCGCTTAACCGGCAGAAGGTTCTGTG	Reverse primer to clone *ftsN*$^{61-105}$ into pJL135

Supplementary Table 4. Summary of different FtsN fusions.

**Construction**	**Insertion site**	**Complementation**	**Doubling time (hr)**	**Localization**
MG1655[a]	N.A.[b]	N.A.[b]	1.7 ± 0.1	Midcell
mEos3.2-FtsN	N-terminus	Yes	N.D.[c]	Midcell
mNG-FtsN	N-terminus	Yes	1.5 ± 0.1	Midcell
P12-mNG-A13	Within FtsN^{Cyto}	Yes	1.8 ± 0.1	Midcell, weak
N28-mNG-L29		Partially	2.5 ± 0.3	Midcell, very weak
E60-mNG-E61	Between FtsN^{TM} and FtsN^{E}	Yes	1.5 ± 0.1	Midcell
K69-mNG-V70		Yes	1.5 ± 0.1	Midcell
Q113-mNG-L114	Between FtsN^{E} and FtsN^{SPOR}	Yes	2.0 ± 0.1	Midcell & Cell pole
Q124-mNG-M125		Yes	1.6 ± 0.1	Midcell & Cell pole
Q151-mNG-T152		Yes	1.6 ± 0.1	Midcell & Cell pole
Q182-mNG-T183		Yes	1.5 ± 0.1	Midcell & Cell pole
Q212-mNG-T213		Yes	1.6 ± 0.2	Midcell & Cell pole
FtsN-mNG	C-terminus	Yes	1.4 ± 0.1	Midcell & Cell pole

All the experiments were performed with cells grown in M9-glucose minimal medium.

[a] This is MG1655 wild-type strain. Localization data was from the immunostaining fluorescence images.

[b] N.A. not applicable.

[c] N.D. not measured.

Supplementary Table 5. FtsN-ring and FtsZ-ring dimension measurements.

Strains	Deconvolved ring width FWHM (nm)[b]	Deconvolved ring thickness FWHM (nm)[b]	n^c
mEos3.2-FtsN	86 ± 3	51 ± 4	72
FtsZ-mEos3.2 [a]	84 ± 2	47 ± 2	103

All the experiments were performed with cells grown in M9-glucose minimal medium.
[a] FtsZ-mEos3.2 data was from a previous work[4].
[b] The FWHM was deconvolved as previously described[21], which allows comparison of dimensions obtained with different spatial resolutions.
[c] *n* is the number of cells used in each measurement.

Supplementary Table 6. Comparison of the moving speed across different divisome proteins.

Proteins[b]	Imaging Modality	$P_1\_V_1$ (%)[c]	V_1 (nm s^{-1})[c]	V_2 (nm s^{-1})[c]
FtsN	TIRF	100	8.7 ± 0.2	N.A.[a]
	SMT	100	9.5 ± 0.2	N.A.[a]
FtsW	SMT	63.6 ± 7.6	9.4 ± 0.3	37.8 ± 6.1
FtsI	SMT	53.9 ± 19.9	9.8 ± 1.1	31.2 ± 5.6
FtsZ	TIRF	0	N.A.[a]	28.0 ± 1.2

All the experiments were performed with cells grown in M9-glucose minimal medium.

[a] N.A. not applicable

[b] FtsN data is from this study, where TIRF data is the combination of TIRF and TIRF-SIM imaging of the mNG-FtsN fusion (Strain 4564 in Supplementary Table 1), SMT data is from SMT imaging of the FtsN-Halo^{SW} fusion (Strain 5234 in Supplementary Table 1). FtsW data is from SMT imaging of a FtsW-RFP fusion in a previous study[5]. FtsI data is from SMT imaging of an RFP-FtsI fusion from a previous study[5]. FtsZ data is from TIRF imaging of an FtsZ-GFP fusion in a previous study[6].

[c] Speeds of FtsN (V_1) and FtsZ (V_2) from the TIRF data were calculated as the average of the absolute speeds. Errors are the *s.e.m.* with $n > 200$. Percentage ($P_1\_V_1$), speed (V_1) of the slow-moving population and speed (V_2) of the fast-moving population from the SMT data of FtsN, FtsW, and FtsI are obtained from one-population (FtsN) or two-population (FtsW, FtsI) free-float fitting of 200 CDF curves bootstrapped from three independent experiments. Errors are the standard deviations of the fitted parameters.

Supplementary Table 7. FtsN dynamics in cells with different FtsZ treadmilling speeds.

FtsZ mutants	FtsZ speed[a] (nm s^{-1}) (N_Z)	*P*_moving[b] (%) (N_{all})	FtsN speed[b] (nm s^{-1})	*T*_moving[c] (s) (N_m)	*T*_stationary[c] (s) (N_s)
WT	28.0 ± 1.2 (182)	44.0 ± 1.3 (161)	9.6 ± 0.4	16.4 ± 1.1 (71)	32.9 ± 3.8 (90)
E238A	23.8 ± 2.9 (37)	43.1 ± 1.7 (149)	10.5 ± 0.4	15.6 ± 1.6 (64)	20.8 ± 1.8 (85)
E250A	17.4 ± 1.6 (41)	43.9 ± 1.6 (285)	9.3 ± 0.3	19.3 ± 1.2 (125)	32.1 ± 2.5 (160)
D269A	14.3 ± 1.3 (32)	41.8 ± 1.5 (219)	9.4 ± 0.3	17.9 ± 1.1 (92)	34.3 ± 3.0 (127)
G105S	9.7 ± 1.0 (35)	42 .5 ± 1.8 (189)	10.0 ± 0.4	14.4 ± 0.8 (80)	37.8 ± 4.4 (109)

All the experiments were performed with cells grown in M9-glucose minimal medium.

[a] FtsZ treadmilling speeds were calculated as the average of the absolute speeds from a previous work[6]. Data are presented as mean ± *s.e.m.* N_z is the number of FtsZ kymograph segments.

[b] Percentage of segment number (*P*_moving) and average speed of FtsN molecules spent in directional moving state. Data are presented as mean ± error, where the error is the standard deviation from 200 bootstrap samples pooled from three independent experiments. N_{all} is the number of total track segments.

[c] Average dwell time of FtsN molecules spent in directional moving state (*T*_moving) and stationary state (*T*_stationary). Data are presented as mean ± *s.e.m.* N_m is the number of segments corresponding to a directionally moving molecule. N_s is the number of segments corresponding to a stationary molecule.

Supplementary Table 8. Dynamics of FtsN's cytoplasmic domain mutants.

FtsN mutant	*P*_moving[a] (%) (N_{all})	FtsN speed[a] (nm s^{-1})	*T*_moving[b] (s) (N_m)	*T*_stationary[b] (s) (N_s)
$FtsN^{D5N}$	43.1 ± 2.4 (202)	9.7 ± 0.4	11.4 ± 0.6 (87)	17.7 ± 1.8 (115)
$FtsN^{\Delta Cyto\text{-}TM}$	40.5 ± 1.6 (234)	9.2 ± 0.3	10.5 ± 0.6 (94)	27.7 ± 2.5 (140)

All the experiments were performed with cells grown in M9-glucose minimal medium.
[a] Percentage of segment number (*P*_moving) and average speed of FtsN mutant molecules spent in directional moving state. Data are presented as mean ± error, where the error is the standard deviation from 200 bootstrap samples pooled from three independent experiments. N_{all} is the number of total track segments.
[b] Average dwell time of FtsN mutant molecules spent in directional moving state (*T*_moving) and stationary state (*T*_stationary). Data are presented as mean ± *s.e.m.* N_m is the number of segments corresponding to a directionally moving molecule. N_s is the number of segments corresponding to a stationary molecule.

Supplementary Table 9. FtsN$^{Cyto\text{-}TM}$ dynamics in cells with different FtsZ treadmilling speeds.

FtsZ mutants	FtsZ speed[a] (nm s^{-1}) (N_Z)	*P*_moving[b] (%) (N_{all})	FtsN$^{Cyto\text{-}TM}$ speed[b] (nm s^{-1})	*T*_moving[c] (s) (N_m)	*T*_stationary[c] (s) (N_s)
WT	28.0 ± 1.2 (182)	62.5 ± 1.9 (130)	29.1 ± 1.7	7.5 ± 0.4 (81)	18.4 ± 1.6 (49)
E238A	23.8 ± 2.9 (37)	59.5 ± 3.1 (108)	26.4 ± 1.5	7.7 ± 0.4 (64)	15.6 ± 1.3 (44)
E250A	17.4 ± 1.6 (41)	54.8 ± 3.0 (96)	21.7 ± 1.2	7.1 ± 0.4 (53)	12.8 ± 0.7 (43)
D269A	14.3 ± 1.3 (32)	45.1 ± 3.1 (104)	15.8 ± 0.7	7.3 ± 0.5 (47)	19.5 ± 2.0 (57)
G105S	9.7 ± 1.0 (35)	37.5 ± 2.0 (197)	10.9 ± 0.5	8.5 ± 0.5 (74)	12.5 ± 0.9 (123)

All the experiments were performed with cells grown in M9-glucose minimal medium.

[a] FtsZ treadmilling speeds were calculated as the average of the absolute speeds from previous work[6]. Data are presented as mean ± *s.e.m.* N_Z is the number of FtsZ kymograph segments.

[b] Percentage of segment number (*P*_moving) and average speed of FtsN$^{Cyto\text{-}TM}$ molecules spent in directional moving state. Data are presented as mean ± error, where the error is the standard deviation from 200 bootstrap samples pooled from three independent experiments. N_{all} is the number of total track segments.

[c] Average dwell time of FtsN$^{Cyto\text{-}TM}$ molecules spent in directional moving state (*T*_moving) and stationary state (*T*_stationary). Data are presented as mean ± *s.e.m.* N_m is the number of segments corresponding to a directionally moving molecule. N_s is the number of segments corresponding to a stationary molecule.

Supplementary Table 10. FtsN dynamics under different sPG synthesis conditions.

Genotype	Drug or medium	P_moving[a] (%) (N_{all})	FtsN speed[a] (nm s^{-1})	T_moving[b] (s) (N_m)	T_stationary[b] (s) (N_s)
BW25113	M9-glucose	44.1 ± 2.2 (161)	9.6 ± 0.4	16.4 ± 1.1 (71)	32.9 ± 3.8 (90)
	MTSES, M9-glucose	42.1 ± 1.5 (176)	9.2 ± 0.4	16.2 ± 1.3 (74)	28.3 ± 1.5 (102)
BW25113, *ftsW*^{I302C}	M9-glucose	43.7 ± 1.3 (155)	9.6 ± 0.5	13.8 ± 1.0 (68)	26.3 ± 1.5 (87)
	MTSES, M9-glucose	19.6 ± 1.4 (143)	9.5 ± 0.5	12.7 ± 0.9 (28)	24.1 ± 2.9 (115)
MG1655	M9-glucose	44.9 ± 1.6 (571)	9.4 ± 0.2	14.5 ± 0.7 (256)	27.3 ± 1.3 (315)
	Aztreonam, M9-glucose	10.3 ± 0.9 (760)	7.9 ± 0.5	19.2 ± 1.1 (79)	42.5 ± 1.2 (681)
	Fosfomycin, M9-glucose	9.9 ± 1.8 (374)	9.7 ± 0.5	15.2 ± 0.9 (37)	40.9 ± 1.8 (337)
	1XPBS (4% PFA fixed)	5.2 ± 0.9 (856)	9.1 ± 0.6	15.4 ± 1.0 (44)	73.7 ± 2.3 (812)
MG1655, *ftsI*^{R167S}	M9-glucose	45.8 ± 2.0 (375)	9.4 ± 0.3	16.0 ± 0.7 (173)	18.5 ± 0.7 (202)
	EZRDM	48.9 ± 1.0 (154)	12.3 ± 0.5	13.8 ± 0.5 (75)	14.4 ± 0.8 (79)
	EZRDM + UppS	49.2 ± 1.7 (140)	13.7 ± 0.5	13.2 ± 0.8 (69)	12.8 ± 0.5 (71)

[a] Percentage of segment number (P_moving) and average speed of FtsN molecules spent in directional moving state. Data are presented as mean ± error, where the error is the standard deviation from 200 bootstrap samples pooled from three independent experiments. N_{all} is the number of total track segments.

[b] Average dwell time of FtsN molecules spent in directional moving state (T_moving) and stationary state (T_stationary). Data are presented as mean ± *s.e.m.* N_m is the number of segments corresponding to a directionally moving molecule. N_s is the number of segments corresponding to a stationary molecule.

Supplementary Table 11. The *p*-values of the two-sample Kolmogorov-Smirnov (K-S) test for FtsN and FtsW's directional movement.

Growth condition	*p*		
	Speed (*V*)	Moving dwell time (*T*_moving)	Processive running length (*PL*)
EZRDM	0.214[a]	0.074[a]	0.369[a]
EZRDM + UppS	0.051[a]	0.052[a]	0.108[a]

[a] The *p*-values indicate the distributions are not significantly different from each other ($p > 0.05$).

Supplementary Table 12. Dynamics of FtsN mutants in the superfission strain.

Genotype	Plasmid	*P*_moving[b] (%) (N_{all})	P_1_V_1[c] (%)	V_1[b, c] (nm s^{-1})	V_2[c] (nm s^{-1})	*T*_moving[d] (s) (N_m)	*T*_stationary[d] (s) (N_s)
TB28, *ΔftsN*, *ftsB*[E56A]	$FtsN^{WT}$	44.0 ± 1.8 (285)	100	9.3 ± 0.3	N.A.[a]	14.8 ± 0.8 (125)	31.2 ± 2.0 (160)
	$FtsN^{WYAA}$	11.0 ± 2.1 (252)	100	13.6 ± 1.3	N.A.[a]	10.9 ± 0.7 (28)	21.4 ± 1.4 (224)
	$FtsN^{E}$	62.9 ± 1.2 (202)	29.8 ± 13.7	8.4 ± 1.9	28.8 ± 6.3	9.1 ± 0.5 (127)	14.1 ± 0.9 (75)
TB28, *ΔftsN*, *ftsB*[E56A], *ftsZ*[E250A]	$FtsN^{E}$	55.3 ± 1.1 (217)	40.0 ± 11.3	8.6 ± 1.6	24.1 ± 1.8	9.6 ± 0.5 (120)	18.8 ± 1.1 (97)
TB28, *ΔftsN*, *ftsB*[E56A], *ftsZ*[G105S]		40.8 ± 0.9 (255)	41.5 ± 27.9	8.0 ± 2.5	11.5 ± 3.4	12.5 ± 0.7 (104)	21.6 ± 1.4 (151)

All the experiments were performed with cells grown in M9-glucose minimal medium.

[a] N.A. not applicable

[b] Percentage of segment number (*P*_moving) and average speed (V_1) of FtsN or FtsN mutant molecules spent in directional moving state. Data are presented as mean ± error, where the error is the standard deviation from 200 bootstrap samples pooled from three independent experiments. N_{all} is the number of total track segments.

[c] Percentage (P_1_V_1), speed (V_1) of the slow-moving population and speed (V_2) of the fast-moving population of $FtsN^{E}$ molecules obtained from two-population free-float fitting of CDF curves bootstrapped 200 times from three independent experiments. Errors are the standard deviations of the fitted parameters.

[d] Average dwell time of FtsN or FtsN mutant molecules spent in directional moving state (*T*_moving) and stationary state (*T*_stationary). Data are presented as mean ± *s.e.m.* N_m is the number of segments corresponding to a directionally moving molecule. N_s is the number of segments corresponding to a stationary molecule.

References

1. Yang, J.C., Van Den Ent, F., Neuhaus, D., Brevier, J. & Lowe, J. Solution structure and domain architecture of the divisome protein FtsN. *Mol. Microbiol.* **52**, 651-660 (2004).
2. Coltharp, C., Buss, J., Plumer, T.M. & Xiao, J. Defining the rate-limiting processes of bacterial cytokinesis. *Proc. Natl Acad. Sci. USA* **113**, 1044-1053 (2016).
3. Fu, G. *et al.* In vivo structure of the E. coli FtsZ-ring revealed by photoactivated localization microscopy (PALM). *PloS one* **5**, e12682 (2010).
4. Lyu, Z., Coltharp, C., Yang, X. & Xiao, J. Influence of FtsZ GTPase activity and concentration on nanoscale Z-ring structure in vivo revealed by three-dimensional Superresolution imaging. *Biopolymers* **105**, 725-734 (2016).
5. Yang, X. *et al.* A two-track model for the spatiotemporal coordination of bacterial septal cell wall synthesis revealed by single-molecule imaging of FtsW. *Nat. Microbiol*. **6**, 584-593 (2021).
6. Yang, X. *et al.* GTPase activity-coupled treadmilling of the bacterial tubulin FtsZ organizes septal cell wall synthesis. *Science* **355**, 744-747 (2017).
7. Guyer, M.S., Reed, R.R., Steitz, J.A. & Low, K.B. Identification of a sex-factor-affinity site in E. coli as gamma delta. *Cold Spring Harb. Symp. Quant. Biol.* **45 Pt 1**, 135-140 (1981).
8. Baba, T. *et al.* Construction of *Escherichia coli* K-12 in-frame, single-gene knockout mutants: the Keio collection. *Mol. Syst. Biol.* **2**, 2006 0008 (2006).
9. Arends, S.J. & Weiss, D.S. Inhibiting cell division in *Escherichia coli* has little if any effect on gene expression. *J. Bacteriol.* **186**, 880-884 (2004).
10. Tarry, M. *et al.* The *Escherichia coli* cell division protein and model Tat substrate SufI (FtsP) localizes to the septal ring and has a multicopper oxidase-like structure. *J. Mol. Biol.* **386**, 504-519 (2009).
11. Chen, J.C. & Beckwith, J. FtsQ, FtsL and FtsI require FtsK, but not FtsN, for co-localization with FtsZ during *Escherichia coli* cell division. *Mol. Microbiol.* **42**, 395-413 (2001).
12. Liu, B., Persons, L., Lee, L. & de Boer, P.A. Roles for both FtsA and the FtsBLQ subcomplex in FtsN-stimulated cell constriction in *Escherichia coli*. *Mol. Microbiol.* **95**, 945-970 (2015).
13. McCausland, J.W. *et al.* Treadmilling FtsZ polymers drive the directional movement of sPG-synthesis enzymes via a Brownian ratchet mechanism. *Nat. Commun.* **12**, 609 (2021).
14. Haldimann, A. & Wanner, B.L. Conditional-replication, integration, excision, and retrieval plasmid-host systems for gene structure-function studies of bacteria. *J. Bacteriol.* **183**, 6384-6393 (2001).
15. Weiss, D.S., Chen, J.C., Ghigo, J.M., Boyd, D. & Beckwith, J. Localization of FtsI (PBP3) to the septal ring requires its membrane anchor, the Z ring, FtsA, FtsQ, and FtsL. *J. Bacteriol.* **181**, 508-520 (1999).
16. Jones-Carson, J. *et al.* Nitric oxide disrupts bacterial cytokinesis by poisoning purine metabolism. *Sci. Adv.* **6**, eaaz0260 (2020).
17. Wissel, M.C. & Weiss, D.S. Genetic analysis of the cell division protein FtsI (PBP3): amino acid substitutions that impair septal localization of FtsI and recruitment of FtsN. *J. Bacteriol.* **186**, 490-502 (2004).
18. Arends, S.J. *et al.* Discovery and characterization of three new *Escherichia coli* septal ring proteins that contain a SPOR domain: DamX, DedD, and RlpA. *J. Bacteriol.* **192**, 242-255 (2010).

19. Williams, K.B. *et al.* Nuclear magnetic resonance solution structure of the peptidoglycan-binding SPOR domain from *Escherichia coli* DamX: insights into septal localization. *Biochemistry* **52**, 627-639 (2013).
20. Buss, J. *et al.* A multi-layered protein network stabilizes the *Escherichia coli* FtsZ-ring and modulates constriction dynamics. *PLoS Genet.* **11**, e1005128 (2015).
21. Coltharp, C., Yang, X. & Xiao, J. Quantitative analysis of single-molecule superresolution images. *Curr. Opin. Struct. Biol.* **28**, 112-121 (2014).

Uncropped scans of all blots and gels

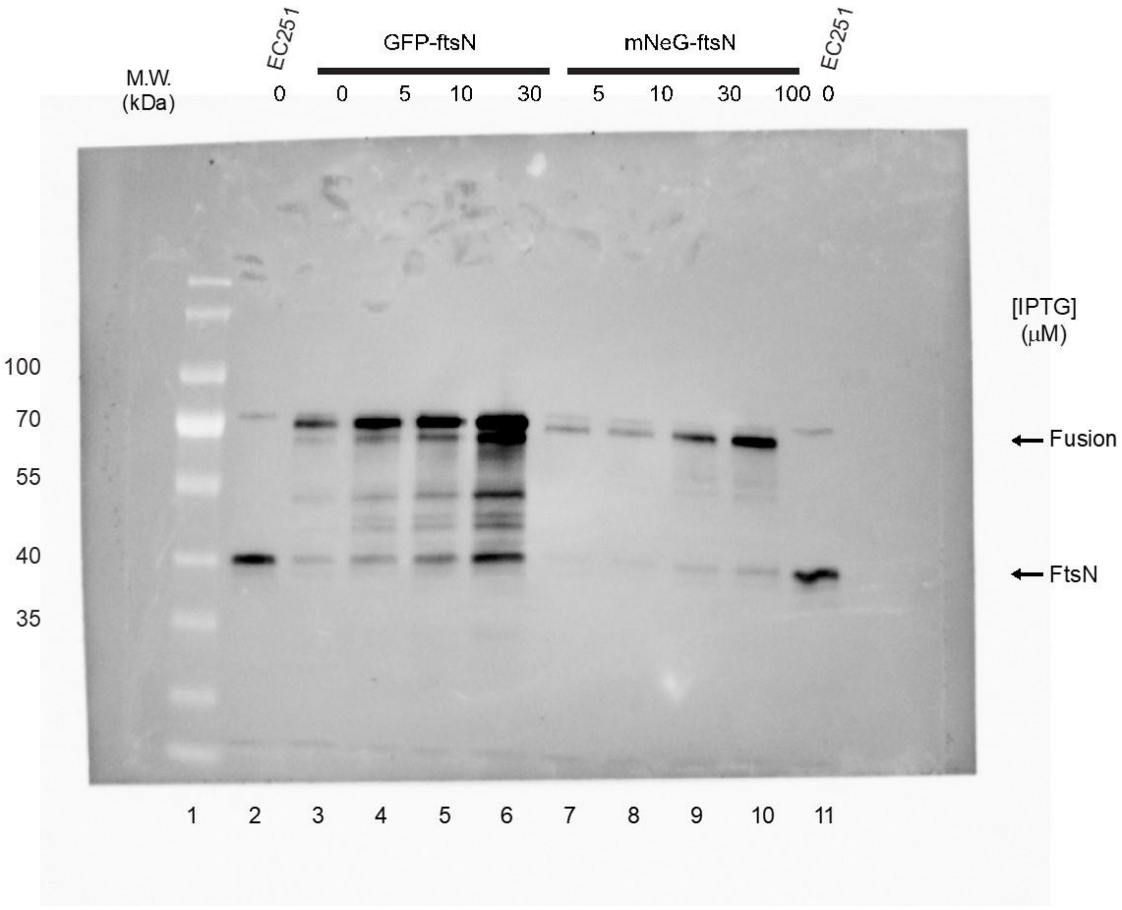

Supplemental Figure 4A

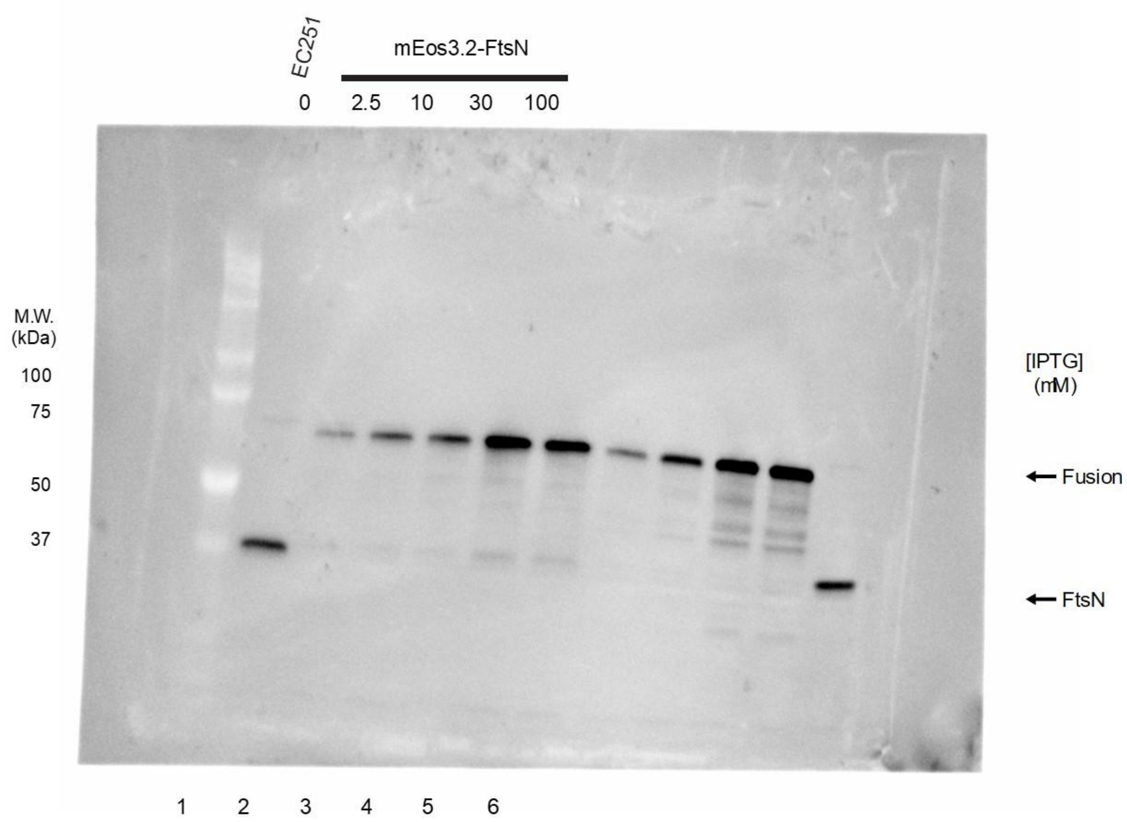

Supplemental Figure 4B

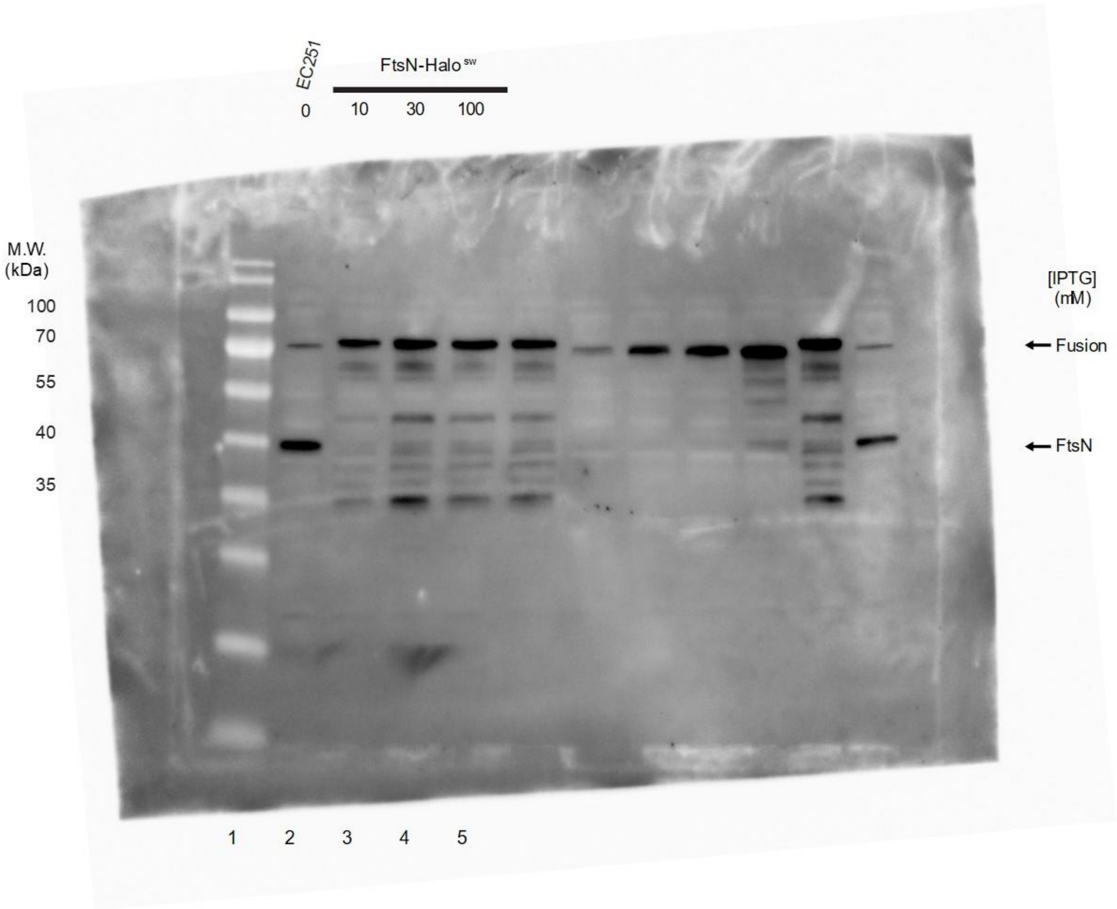

Supplemental Figure 4C

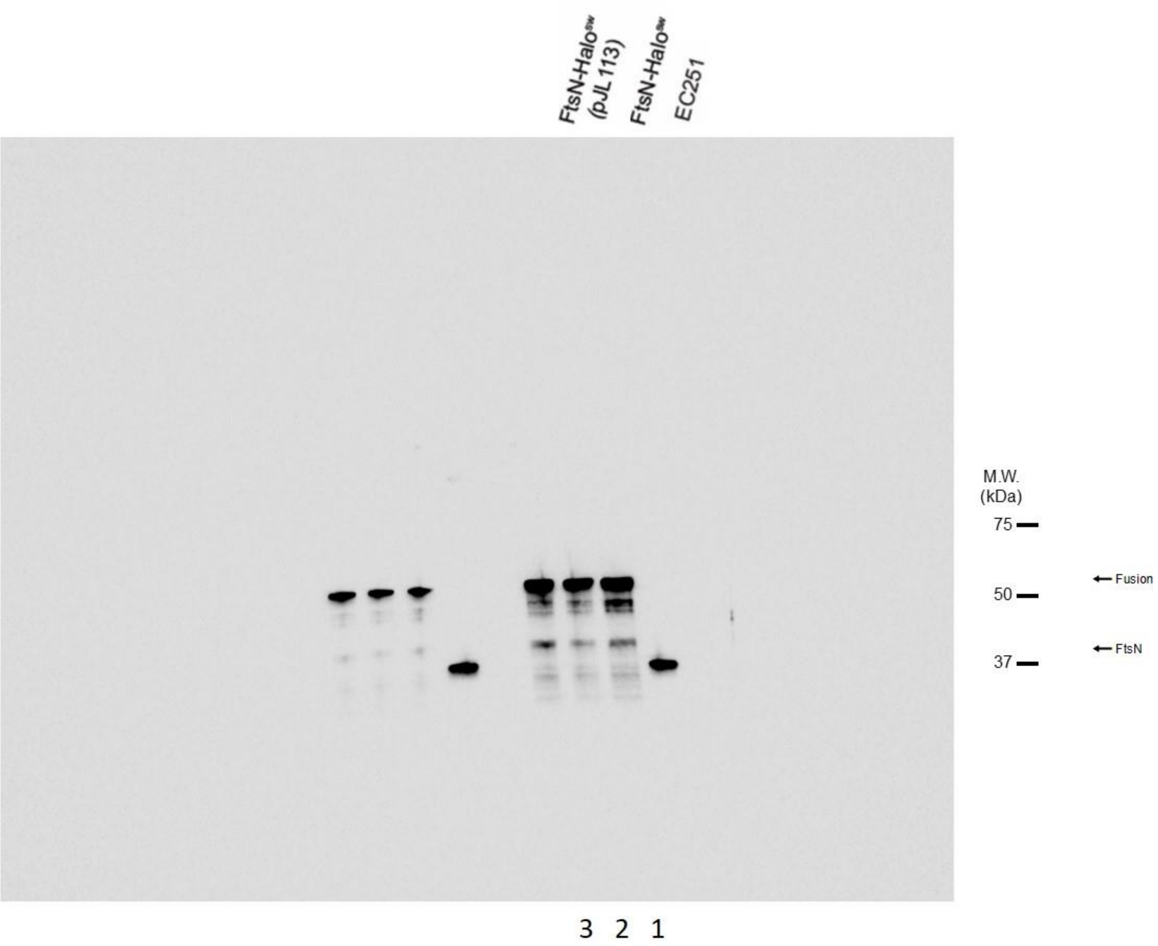

Supplemental Figure 4D

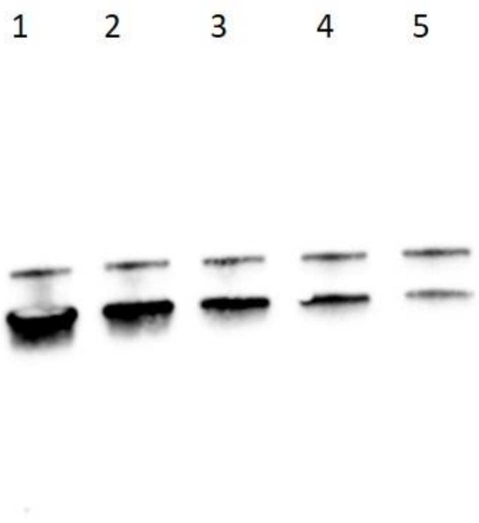

Supplemental Figure 5A

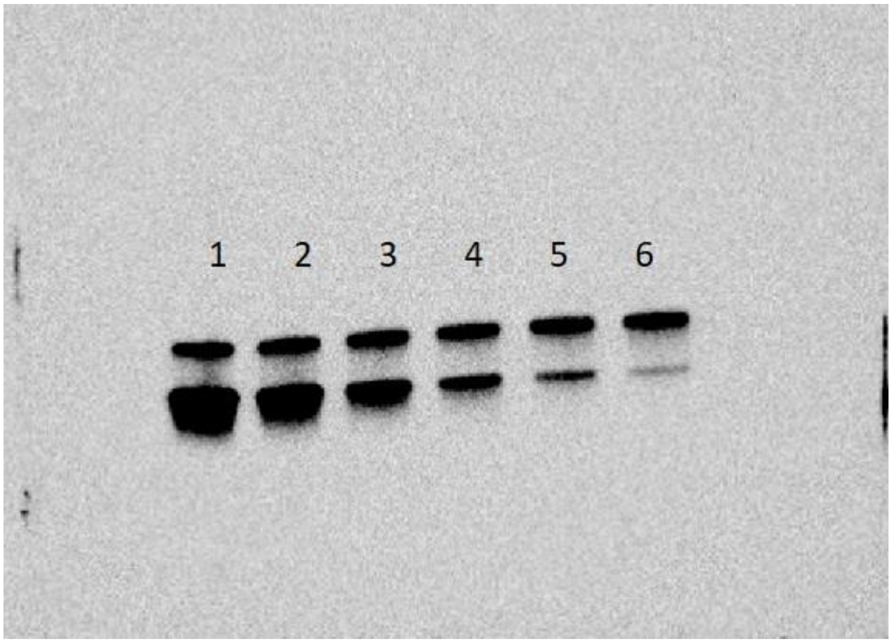

Supplemental Figure 5B

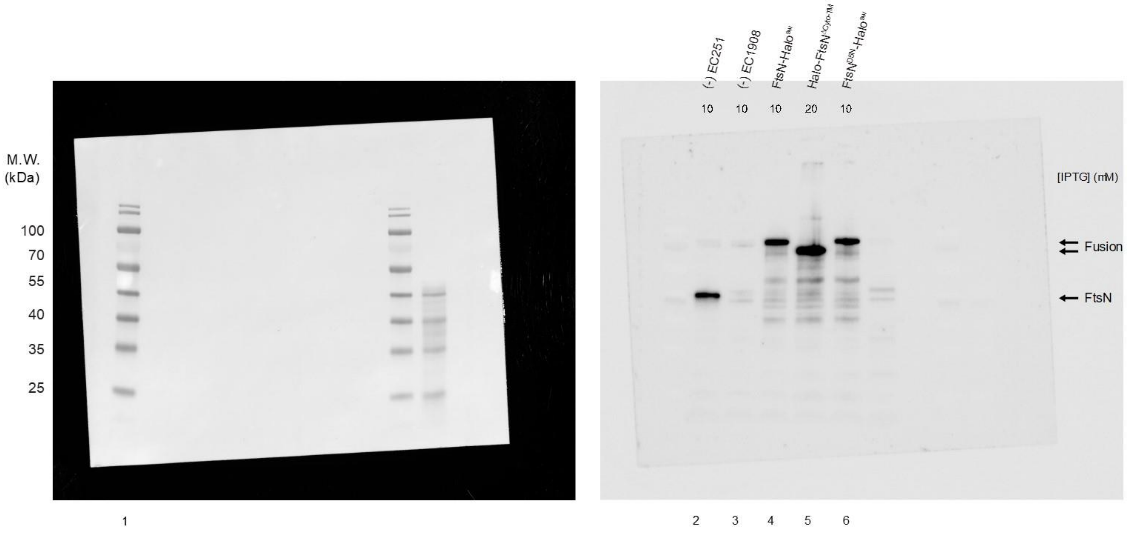

Supplemental Figure 11A
