# Supplementary material for: FtsN maintains active septal cell wall synthesis by forming a processive complex with the septum-specific peptidoglycan synthases in E. coli
Source: Nat Commun. 2022 Sep 30;13:5751. doi: 10.1038/s41467-022-33404-8 (PMC9525312; doi:10.1038/s41467-022-33404-8)
Supplement: Supplementary file 3 — Description of Additional Supplementary Information files [file 41467_2022_33404_MOESM3_ESM.pdf]

Description of Additional Supplementary Information

Supplementary Movie 1. Time-lapse FRAP imaging of GFP-FtsN fusion cells.
Half of the ring in the cell on the right was bleached (cyan arrow). The cell on the left served as a control (yellow arrowhead). Images were acquired every 1 s for 150 s after photobleaching, with a 50-ms exposure time. Scale bars, 500 nm.

Supplementary Movie 2. Time-lapse TIRF-SIM imaging of a mNG-FtsN fusion cell.
Images were acquired every 1 s for 40 s, with a 50-ms exposure time. Scale bars, 500 nm.

Supplementary Movie 3. SMT imaging of a cell with an immobile JF646-FtsN-Halo^{SW} molecule.
Images were acquired every 1 s for 150 s, with a 100-ms exposure time. Scale bars, 500 nm.

Supplementary Movie 4. SMT imaging of a cell with a processively moving JF646-FtsN-Halo^{SW} molecule.
Images were acquired every 1 s for 150 s, with a 100-ms exposure time. Scale bars, 500 nm.

Supplementary Movie 5. SMT imaging of a cell with a JF646-FtsN-Halo^{SW} molecule switching between the stationary state and moving state with different speeds.
Images were acquired every 1 s for 150 s, with a 100-ms exposure time. Scale bars, 500 nm.
