# Supplementary material for: FtsN maintains active septal cell wall synthesis by forming a processive complex with the septum-specific peptidoglycan synthases in E. coli
Source: Nat Commun. 2022 Sep 30;13:5751. doi: 10.1038/s41467-022-33404-8 (PMC9525312; doi:10.1038/s41467-022-33404-8)
Supplement: Supplementary file 9 — Reporting Summary [file 41467_2022_33404_MOESM9_ESM.pdf]

nature portfolio

Corresponding author(s): Jie Xiao, David Weiss

Last updated by author(s): Sep 1, 2022

Reporting Summary

Nature Portfolio wishes to improve the reproducibility of the work that we publish. This form provides structure for consistency and transparency in reporting. For further information on Nature Portfolio policies, see our Editorial Policies and the Editorial Policy Checklist.

Statistics

For all statistical analyses, confirm that the following items are present in the figure legend, table legend, main text, or Methods section.

n/a	Confirmed	
☐	☒	The exact sample size (n) for each experimental group/condition, given as a discrete number and unit of measurement
☐	☒	A statement on whether measurements were taken from distinct samples or whether the same sample was measured repeatedly
☐	☒	The statistical test(s) used AND whether they are one- or two-sided<br>*Only common tests should be described solely by name; describe more complex techniques in the Methods section.*
☒	☐	A description of all covariates tested
☒	☐	A description of any assumptions or corrections, such as tests of normality and adjustment for multiple comparisons
☐	☒	A full description of the statistical parameters including central tendency (e.g. means) or other basic estimates (e.g. regression coefficient) AND variation (e.g. standard deviation) or associated estimates of uncertainty (e.g. confidence intervals)
☐	☒	For null hypothesis testing, the test statistic (e.g. F, t, r) with confidence intervals, effect sizes, degrees of freedom and P value noted<br>*Give P values as exact values whenever suitable.*
☒	☐	For Bayesian analysis, information on the choice of priors and Markov chain Monte Carlo settings
☒	☐	For hierarchical and complex designs, identification of the appropriate level for tests and full reporting of outcomes
☒	☐	Estimates of effect sizes (e.g. Cohen's d, Pearson's r), indicating how they were calculated

Our web collection on statistics for biologists contains articles on many of the points above.

Software and code

Policy information about availability of computer code

Data collection	Custom journals were written in Metamorph 7.8.13.0 software to acquire time-stream SMLM images, time-lapse FRAP, TIRF, and single-molecule images. DeltaVision™ OMX SR software on the GE OMX-SR Super-Resolution Microscope was used to acquire time-lapse TIRF-SIM images.
Data analysis	ImageJ software (v.1.52p) and the ThunderSTORM plug-in were used to identify the fluorophore localization for single-molecule tracking analysis. Single-molecule tracking and post data processing were done with custom programming codes written in Matlab. Codes are uploaded to the Xiao Lab's GitHub and linked to Zenodo (https://zenodo.org/record/4306646).

For manuscripts utilizing custom algorithms or software that are central to the research but not yet described in published literature, software must be made available to editors and reviewers. We strongly encourage code deposition in a community repository (e.g. GitHub). See the Nature Portfolio guidelines for submitting code & software for further information.

Data

Policy information about availability of data

All manuscripts must include a data availability statement. This statement should provide the following information, where applicable:

- Accession codes, unique identifiers, or web links for publicly available datasets
- A description of any restrictions on data availability
- For clinical datasets or third party data, please ensure that the statement adheres to our policy

The authors declare that all other data supporting the findings of this study are available within the paper and its supplementary information files. Source data are

provided with this paper.

Human research participants

Policy information about studies involving human research participants and Sex and Gender in Research.

Reporting on sex and gender	N.A.
Population characteristics	N.A.
Recruitment	N.A.
Ethics oversight	N.A.

Note that full information on the approval of the study protocol must also be provided in the manuscript.

Field-specific reporting

Please select the one below that is the best fit for your research. If you are not sure, read the appropriate sections before making your selection.

☒ Life sciences ☐ Behavioural & social sciences ☐ Ecological, evolutionary & environmental sciences

For a reference copy of the document with all sections, see nature.com/documents/nr-reporting-summary-flat.pdf

Life sciences study design

All studies must disclose on these points even when the disclosure is negative.

Sample size	No specific sample size calculation or statistic method was made. The sample size varied in different experiments described below and in the manuscript.<br>For SMLM imaging experiment, 72 E. coli cells were imaged and analyzed from three biological independent experiments to get the ring structure. The cell diameter data showed the imaged cells covered the whole cell division period, which means the sample size is sufficient.<br>For fluorescence recovery after photobleaching (FRAP) experiment, 58 E. coli cells were imaged and analyzed from two biological independent experiments to get the recovery time after photobleaching. The errors of the averaged recovery curve is way much smaller than the recovery changes, which means the sample size is sufficient.<br>For TIRF or TIRF-SIM experiment, at least 90 E. coli cells were imaged and analyzed from five or four biological independent experiments to get the cluster dynamics. The cluster movement speeds showed a good normal distribution, which means the sample size is sufficient.<br>For growth curve measurements and immunoblots, two to three repeats were used to get the average results. The results from the repeats were very close to each other, which means the sample size is sufficient.<br>For single-molecule tracking experiments, at least 100 trajectories (the numbers are listed in the Supplementary tables) were monitored to reconstruct the distribution and cumulative probability density function for fitting. The errors of mean were clearly listed in the main text and supplementary tables. The single-molecule movement speeds showed a good normal distribution, which means the sample size is sufficient.
Data exclusions	Fluorescence spots with an intensity over 3 sigma of the peak intensity were removed from analysis since they likely include more than one molecule. Short trajectories (shorter than 5 time points) of a single molecule are excluded because they cannot be accurately classify or quantify to moving or free diffusion molecules based on our simulation.
Replication	Two to four biological replicates were done by culturing different source colonies of E. coli from the same genotype. The cell growth, drug treatment, and imaging experiments were completed at different days with other conditions such as temperature or cell density kept as similar as possible. The exact replicate numbers for each experiments were listed in the main text and supplementary file. All attempts at replication were successful.
Randomization	Samples were not allocated to groups.
Blinding	For the SMLM, FRAP, TRIF, and TIRF-SIM experiments, the data collection were completely blinded. However, cells with obvious ring in the middle were chosen to analyze since the rings were the object of analysis. For the single-molecule tracking, the data collection is intrinsically blinded since the information from single molecules was randomly collected and not be able to choose with bias. Data analysis was not blinded considering the analysis pipeline is more computer-based and standard.

Reporting for specific materials, systems and methods

We require information from authors about some types of materials, experimental systems and methods used in many studies. Here, indicate whether each material, system or method listed is relevant to your study. If you are not sure if a list item applies to your research, read the appropriate section before selecting a response.

Materials & experimental systems

n/a	Involved in the study	
☐	☒	Antibodies
☒	☐	Eukaryotic cell lines
☒	☐	Palaeontology and archaeology
☒	☐	Animals and other organisms
☒	☐	Clinical data
☒	☐	Dual use research of concern

Methods

n/a	Involved in the study	
☒	☐	ChIP-seq
☒	☐	Flow cytometry
☒	☐	MRI-based neuroimaging

Antibodies

Antibodies used	Primary antibody: polyclonal rabbit anti-FtsN sera (From Dr. David S. Weiss). Secondary antibody: HRP goat anti-rabbit (ThermoFisher, 31460)
Validation	Anti-FtsN has been published (Wissel M C, Weiss D S. Genetic analysis of the cell division protein FtsI (PBP3): amino acid substitutions that impair septal localization of FtsI and recruitment of FtsN. Journal of bacteriology, 2004, 186(2): 490-502.
